# Tertiary structure assessment at CASP15

**DOI:** 10.1002/prot.26593

**Published:** 2023-09-25

**Authors:** Adam J. Simpkin, Shahram Mesdaghi, Filomeno Sánchez Rodríguez, Luc Elliott, David L. Murphy, Andriy Kryshtafovych, Ronan M. Keegan, Daniel J. Rigden

**Affiliations:** ^1^ Department of Biochemistry, Cell and Systems Biology Institute of Structural, Molecular and Integrative Biology, University of Liverpool Liverpool UK; ^2^ Computational Biology Facility, MerseyBio, University of Liverpool Liverpool UK; ^3^ Life Science, Diamond Light Source, Harwell Science and Innovation Campus Oxfordshire UK; ^4^ Department of Chemistry, York Structural Biology Laboratory University of York York UK; ^5^ Genome Center, University of California Davis California USA; ^6^ UKRI‐STFC, Rutherford Appleton Laboratory, Research Complex at Harwell Didcot UK

**Keywords:** CASP15, machine learning, molecular replacement, protein modelling, protein structure prediction, structural bioinformatics

## Abstract

The results of tertiary structure assessment at CASP15 are reported. For the first time, recognizing the outstanding performance of AlphaFold 2 (AF2) at CASP14, all single‐chain predictions were assessed together, irrespective of whether a template was available. At CASP15, there was no single stand‐out group, with most of the best‐scoring groups—led by PEZYFoldings, UM‐TBM, and Yang Server—employing AF2 in one way or another. Many top groups paid special attention to generating deep Multiple Sequence Alignments (MSAs) and testing variant MSAs, thereby allowing them to successfully address some of the hardest targets. Such difficult targets, as well as lacking templates, were typically proteins with few homologues. Local divergence between prediction and target correlated with localization at crystal lattice or chain interfaces, and with regions exhibiting high B‐factor factors in crystal structure targets, and should not necessarily be considered as representing error in the prediction. However, analysis of exposed and buried side chain accuracy showed room for improvement even in the latter. Nevertheless, a majority of groups produced high‐quality predictions for most targets, which are valuable for experimental structure determination, functional analysis, and many other tasks across biology. These include those applying methods similar to those used to generate major resources such as the AlphaFold Protein Structure Database and the ESM Metagenomic atlas: the confidence estimates of the former were also notably accurate.

## 1 INTRODUCTION

For nearly 30 years, the Critical Assessment of Structure Prediction (CASP) experiments have monitored, assessed, and incentivized developments in protein structure prediction.^1^, ^2^ Every 2 years, predicting groups are invited to model protein sequences in advance of their experimental structures—be they determined by x‐ray crystallography, cryo‐electron microscopy, or nuclear magnetic resonance—becoming publicly available. Independent groups then assess performance using standardized metrics and statistical models in order to rank groups by performance at each event. More importantly, the exercise also serves to publicize progress and stand‐out methods to the broader communities who benefit from protein modeling, and to point to areas requiring further improvement.

Central to the CASP endeavor since the beginning has been assessment of structural modeling of single protein chains. Even in the earliest CASP exercises, simpler targets—those for which a homologous structure could be identified in the Protein Data Bank^3^—were modeled quite well. In contrast, ab initio modeling (aka de novo or template‐independent modeling) has seen dramatic progress from very poor performance in the early days,^4^ via increasingly sophisticated fragment assembly methods that made good models of small proteins^5^ to the modern era of Machine Learning and especially Deep Learning.^6^ At CASP14, AlphaFold 2 (AF2) emerged as the top‐performing method, by some distance, in both the template‐based^7^ and ab initio^8^ categories. Importantly, the performance on hard targets was close to that seen on easier targets^2^ rendering unnecessary the twin‐track assessment. Hence, at CASP15, the organizers united the previous two categories into one assessment of modeling of single protein chains.

The results of the CASP15 assessment of single chain modeling are presented here. There were submissions from 132 groups for 112 evaluation units (EUs) deriving from 77 single chain targets. We evaluated the performance of the 118 groups, which had submitted models for at least 10 of the 112 EUs. In contrast to CASP14, there was no single stand‐out group: many groups performed very well on a majority of targets with the leading groups distinguished by their ability to tackle the hardest targets. Most of the best‐performing groups used AF2 in one way or another. The two submissions using protein Language Model (pLMs) suggest that such methods are not yet competitive with those using Multiple Sequence Alignments (MSAs), and claimed benefits for targets with no or few homologues^9^, ^10^ were not apparent on the CASP15 targets. Nevertheless, many models, including those derived by pLM‐based methods can, with appropriate and sometimes essential editing, solve most crystal structures.

## 2 MATERIALS AND METHODS

### 2.1 Target definition

The procedures for processing full‐length CASP15 targets into EUs are described in detail elsewhere.^11^ Briefly, protein chains were split into compact structural units (here referred to simply as domains) using the results of several different analytic methods, and also considering similarities to other protein structures. These domains were combined into EUs where a majority of groups successfully captured their relative orientation. Where, on the other hand, groups predicted the individual structural units well but a majority failed to predict their packing, the individual structural domains were retained as EUs. Overall, 112 EUs were derived from 77 tertiary structure prediction targets. Three EUs—T1114s1‐D2, T1157s1‐D2, and T1157s1‐D3—were not evaluated because of the low resolution of the cryo‐EM maps in their local areas.

As in previous CASPs, EUs were assigned to target difficulty categories: TBM (template‐based modeling, easy or hard), FM (free modeling), and the TBM/FM overlap category. Unlike previous CASPs, this procedure was done automatically using methods designed to recapitulate, as far as possible, previous assignments that had significant manual input.^11^ Ultimately, there were 47 EUs in the TBM‐easy class, 15 TBM‐hard, 8 TBM/FM, and 39 in the FM category. This last number is significantly larger than seen in recent CASPs.

### 2.2 Scoring and ranking

Following the practice established by previous CASPs, the group ranking was done using a composite score including metrics relating to global fold correctness, main chain quality, side chain accuracy, and the accuracy of the confidence estimates. Z‐scores were employed to make all measures dimensionless and to represent relative, rather than absolute, performance across all measures in a uniform way. The CASP15 score was modified from the CASP14 predecessor to include reLLG values. The CASP14 score^7^ was:

$$S_{{\text{CASP}}_{14}}=\begin{array}{l} (\frac{1}{16}\left(Z_{\text{LDDT}}+Z_{{\mathrm{CAD}}_{\mathrm{aa}}}+Z_{\mathrm{SG}}+Z_{\text{sidechain}}\right)\\ +\;\frac{1}{12}\left(Z_{\text{MolPrb}-\text{clash}}+Z_{\text{backbone}}+Z_{\text{DippDiff}}\right)\\ +\;\frac{1}{4}\left(Z_{\mathrm{GDT}-\mathrm{HA}}+Z_{\mathrm{ASE}}\right)) \end{array}$$

Here, the set of metrics in the first parenthesis focuses on local and side chain quality: *LDDT* is the local Difference Distance Test that evaluates the agreement between the all‐atom distance maps of target and model,^12^ *CADaa* is the all atom variant of the CAD score looking at residue contact surface areas,^13^ *Sphere‐Grinder* (*SG*) measures how well the model captures the local atomic environments of each residue,^14^ and *sidechain* refers to one of the two torsion angle deviation metrics introduced for template‐based model assessment at CASP13.^15^ The second group focuses more on main chain quality. It includes *MolPrb‐clash*, which refers to the number of serious atom clashes detected by MolProbity^16^ (the calculation also involves side chains), *backbone*, the second, main chain‐focused of Croll et al.'s torsion angle deviation metrics,^15^ and *DipDiff*, which measures interatomic distances involving Cα and O atoms between neighboring residues and compares them between target and model.^17^ In the third group are *GDT_HA*, the high‐accuracy variant of the Global Distance Test—Total Score (GDT_TS), which measures global fold accuracy^18^ and *ASE*, the Accuracy Self Estimate, measuring the correlation between error estimates and actual model errors.

For CASP15, we implemented two modifications to the ranking score. First, the ASE measure was calculated on atomic predicted LDDT values (pLDDT) instead of predicted coordinate errors in Ångstroms,^19^ and second, the reLLG measure^20^ was added to the scoring. The change in the ASE calculation was dictated by the change in the prediction requirements for CASP15, where predictors were asked to estimate accuracy of atoms' placements in a model in terms of pLDDT and not the distance as in CASP14. The inclusion of the reLLG is rationalized by its modest correlation with other elements of the ranking score (the highest pairwise correlation coefficient was 0.69 with GDT_HA) and the importance of protein modeling for its widely‐used downstream application in the experimental protein crystallography. Conceptually, the reLLG is a coordinates‐only score predicting the usefulness of a model for Molecular Replacement (MR).^20^ We included it in the ranking formula with the same weight as GDT_HA and ASE.

The CASP15 score was:

$$S_{{\text{CASP}}_{14}}=\begin{array}{l} (\frac{1}{16}\left(Z_{\text{LDDT}}+Z_{{\mathrm{CAD}}_{\mathrm{aa}}}+Z_{\mathrm{SG}}+Z_{\text{sidechain}}\right)\\ +\;\frac{1}{12}\left(Z_{\text{MolPrb}-\text{clash}}+Z_{\text{backbone}}+Z_{\text{DippDiff}}\right)\\ +\;\frac{1}{4}\left(Z_{\mathrm{GDT}-\mathrm{HA}}+Z_{\mathrm{ASE}}+Z_{\text{reLLG}}\right)) \end{array}$$

We considered changing the assessment of side chain accuracy. However, additional potential metrics were found to correlate too strongly to the existing side chain torsion angle deviation metric to justify inclusion. For example, the GDC_SC measure, a Global Distance Calculation for side chains, had a correlation coefficient of 0.95 with the GDT_HA metric that was already part of the composite score. Similarly, the Average Absolute Accuracy (AAA) measurement from the SCWRL4 package^21^ had a correlation coefficient of −0.96 with the existing side chain torsion angle score.

As mentioned, z‐scores were used instead of raw scores and, as is also customary at CASP, the calculations proceeded in two rounds. In the first, models scoring below an initial z‐score of −2 were considered as outliers and excluded. Z‐scores were then recalculated, but only positive z‐scores included in the ranking calculations. In this way, groups who, perhaps through more speculative and experimental methods, produced a few dramatically poor models were not prevented from having consistently good performance recognized elsewhere. Again as previously, the final rankings were based on sums of z‐scores in order to reward groups performing well across all targets. Finally, it is to be noted that the composite scoring addresses previous concerns that models may capture an overall global fold correctly, but perform poorly in other regards.^15^ Nevertheless, since the accuracy of the fold is the primary consideration, relative performance of groups on GDT_HA alone was also examined.

Calculations were done using a modified version of the code repository created for CASP14 by Joana Pereira and are available at https://github.com/hlasimpk/CASP15_high_accuracy.

MSA depth was measured as Neff/length with data kindly provided by Claudio Mirabello from the National Bioinformatics Infrastructure Sweden at SciLifeLab (doi.org/10.17044/scilifelab.22769996). Calculations were based on alignments that were generated by the public AlphaFold2 server with default parameters and databases (https://github.com/clami66/AF_server/). Classification of targets using DSSP^22^, ^23^ defined them as all‐α (100% of regular secondary structure was α‐helix), mostly α (>65% α), mixed, mostly β (>65%), or all‐β (100% of regular secondary structure was β‐sheet).

### 2.3 Factors affecting local model quality

In order to determine whether local modeling errors were more likely to be found in the vicinity of intermolecular interfaces, predicted models were analyzed using the procedure described in the CASP14 refinement assessment study.^24^ Only higher quality (GDT_TS > 80) model_1 submissions were included in the analysis so that errors could be considered local, rather than as resulting from overall global poor performance. Error was assessed and compared to structural context at both residue and “region” levels.

1. For residue level analysis, the LGA distance (between the predicted model and experimental structure superimposed using the sequence‐dependent algorithm) was compared for residues contributing to crystal, chain, or domain interfaces or to none of these. Residues were considered at a crystal, chain, or domain interface where they had at least three <10 Å Cα–Cα contacts with residues in neighboring symmetry mates, chains, or domains, respectively.
2. Error regions were defined as follows. A five residue‐window rolling average LGA distance (defined as above) was calculated for each residue in the predicted models. Error regions were then defined as comprising at least three consecutive residues with a rolling LGA average of at least 3 Å. These error regions were then defined as being at a crystal lattice interface, a chain interface or a domain interface if the residues within the region had an average of at least 0.5 residues within a radius of 10 Å in a symmetry mate, another chain or a different domain, respectively.^24^ Again, distances were measured between Cα atoms.

To assess the relationship between B‐factors and local error, for selected groups, residues in higher‐quality (GDT_TS > 80) model_1 submissions were analyzed. Within each target, residue B‐factors were first normalized. All residues from all targets were then combined and residues placed into 10 equally sized bins according to normalized B‐factors.

### 2.4 Side chain assessment

SCRWL4's AAA sidechain score^21^ measures the percentage of the model's χ‐angles for each residue that are within 40° of their corresponding angles in the reference structure. A score was calculated for each residue and then averaged over surface and non‐surface side chains for the top‐scoring model (by GDT_HA) for each target. To define surface and non‐surface residues, the Shrake–Rupley algorithm^25^ was used. Prior to the definition of EUs, the solvent accessibility of each target residue was calculated, and a residue was considered part of the surface if its solvent accessibility was greater than 20%.^26^

In addition, the torsion angle deviation metrics for side chain and main chain^15^ were plotted against each other as contour maps in order to assess the dependence of side chain placement accuracy on main chain modeling.

### 2.5 Scoring against x‐ray crystallographic data and MR

#### 2.5.1 Assessing the models' potential for success in MR

Models were tested against the experimental diffraction data, where available, by calculating LLGs for ideally placed structures and then, for selected groups, by attempting MR. Details for the set of 17 targets with diffraction data are shown in the Table S1. The top submitted model by each group, model_1, was first processed to remove residues with low pLDDT values. The current version of Slice'N'Dice^27^ (SnD) removes residues for which the B‐factor column, here recording pLDDT values, contains a value below 70 for the first atom encountered. Here that means that a few residues may have been discarded where their first atomic pLDDT was less than 70 but the mean across the residue exceeded that value (or kept where the reverse was true). However, since only four groups—namely Bench, Yang, Yang‐Server, and Yang‐Multimer—chose to submit atomic, rather than per‐residue, pLDDTs the impact of this was small. Note that no attempt was made here to correct entries from the handful of groups who apparently did not have pLDDT on the expected 0–100 scale in the B‐factor column. The model was then placed ideally onto the target crystal structure using Gesamt^28^ to do the structural superposition. Models were placed for all copies of the molecule in the asymmetric unit. The positioning of the model(s) was further optimized by using Phaser^29^ to perform rigid refinement. This step also generated a total Log Likelihood Gain (LLG) for the placed model(s). A simple ranking of groups was generated by awarding the group responsible for the best model of a target *x* points, where *x* is the total number of groups having attempted a prediction for 1 or more of the 17 targets. The group producing the model with the second‐best LLG was awarded x‐1 points and so on. An LLG of 60 or more for the placement of the first component in an MR search is considered to be indicative of correct placement.^30^ Models scoring less than this threshold did not receive any points. Groups that did not attempt a prediction for the target received no points. The ranking was on the total number of points across the 17 targets for which diffraction data were available.

The impact of domain splitting on alignment between models and target and resulting LLG values was also explored. Using the slice function of the SnD pipeline in CCP4, models were subjected to splitting into 2–4 rigid regions that might separately fit better to corresponding regions in the target structure. In order to process models of all origins uniformly, CCTBX's^31^ PAE‐based domain decomposition was not used: instead the purely coordinate‐based Birch algorithm from SciKit‐learn^32^ was applied. These domain regions were then placed in the same way as described above, producing LLG values for all components matching the contents of the asymmetric unit.

#### 2.5.2 Full MR

Model 1 from a subset of the top scoring groups from the alignment tests were used in a full MR test for each of the 17 targets. The same model from the ESM‐single‐sequence group was also used in these tests to assess how well models produced using the pLM‐based method would perform in full MR. For this test, the unsplit models were used and subjected to B‐factor conversion and with residues having a pLDDT below 70 removed. Phaser was used to carry out the MR with success measured by the resulting LLG.

The case of T1145 (636 residues) was explored in more detail since the presence of several domains made it likely that predicted models would differ from the conformational state captured in the crystal structure. Determining the structure using a predicted model is further complicated by the presence of two copies in the asymmetric unit. The resolution of the diffraction data is 2.2 Angstroms. SnD was used to attempt a structure solution using search models generated by splitting the model into 2, 3, and 4 pieces. MR was also attempted using the unsplit model. Results were refined with Refmac5^33^ before model rebuilding using Modelcraft.^34^ Success was measured using the Rfactors achieved following the model rebuilding step.

### 2.6 Function prediction

Targets were selected based on their interpretability by structure‐based methods. The selection was based on information given to the CASP predictors in combination with analysis and literature review of the targets. Four enzymes T1146 (a putative peptidoglycan hydrolase with a catalytic triad), T1110 (isocyanide hydratase with a catalytic dyad), T1127 (NATA1 with a catalytic dyad), and T1188 (chitinase with a catalytic triad) were selected to determine the accuracy of modeling of catalytic sites. The fit function of PyMOL was used to calculate all‐atom rmsd values between catalytic sites in predictions and targets. The chemical equivalence of side chains for Asp, Gly, and Tyr on 180° rotations about their Cβ–Cγ bonds was taken into account. Only models with GDT_HA > 30 were considered in the analysis.

Three targets were identified as DNA‐binding proteins: T1153, T1170, and T1151. The three experimental structures were screened for predicted DNA‐binding capability with both DNABIND^35^ and BindUP.^36^ Of the three targets, only T1151 was predicted to be a DNA‐binding protein by either method so only models for T1151 were processed.

## 3 RESULTS

### 3.1 Overall group rankings

**FIGURE 1:**
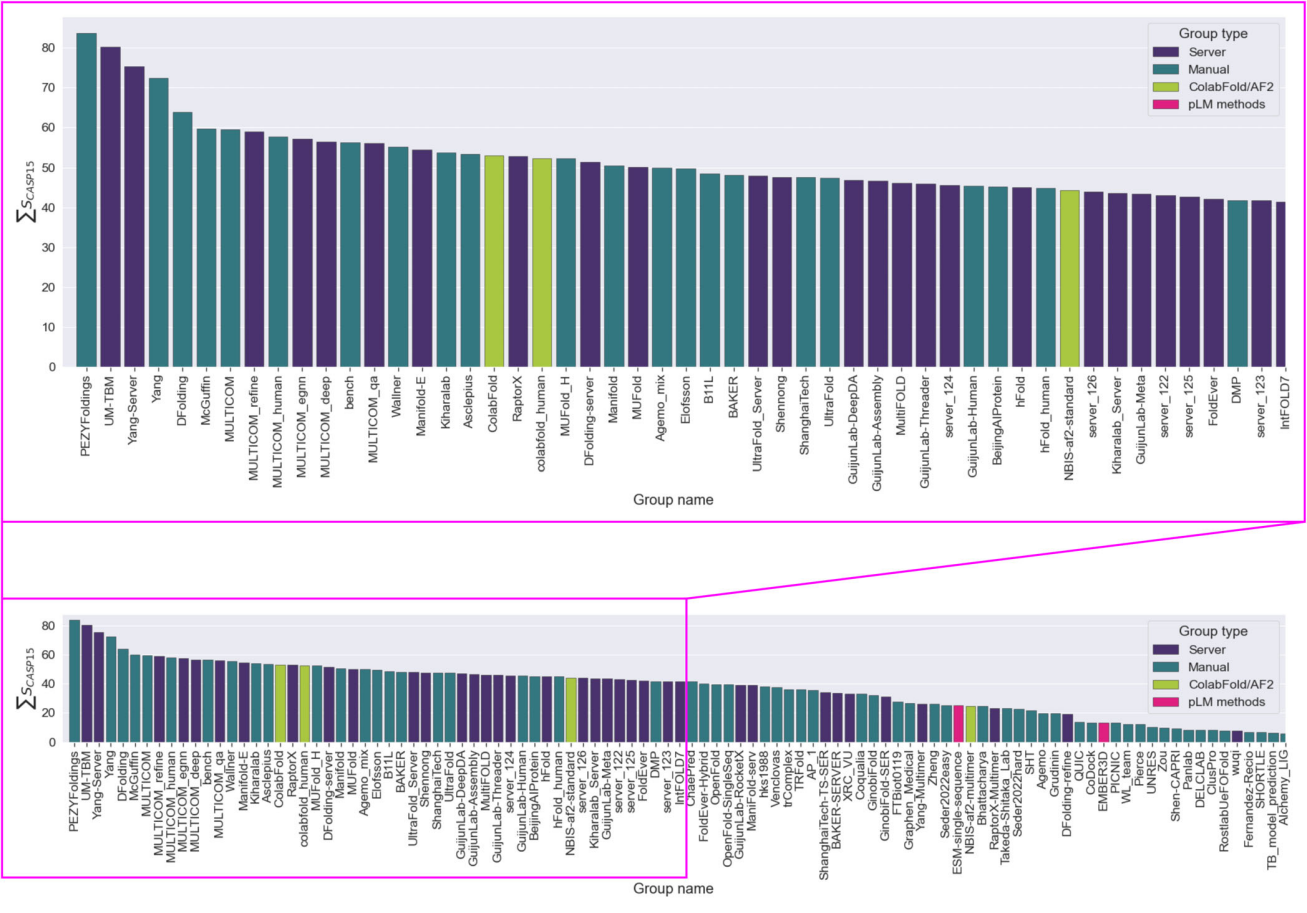
Cumulative group ranking on 109 CASP15 evaluation units. Groups are color‐coded indigo for server, that is, a purely automated modeling protocol, and teal for manual where human intervention is allowed. Pure AF2 comparison runs based on the original DeepMind protocol or its ColabFold version are shown in green. Pink is used for the two groups employing exclusively protein Language Model methods.

Figure 1 shows the CASP15 group ranking, expressed as the sum of the per‐target S_CASP15_ scores, as defined in Materials and Methods. Comparison with the rankings according to the previous CASP formulae (Figure S1) shows very similar results in the top positions. Several interesting conclusions can be drawn. First, automated servers (indigo in Figure 1) are higher in rankings and appear better represented at the top of the performance table in CASP15 than in any previous CASP. Of the top three places in CASP15, two are occupied by servers, which never happened before in any of the prediction categories.

Second, it is clear that the “control” runs of AF2, both in the original DeepMind implementation as of March 2022 (group names NBIS‐AF2‐standard and NBIS‐AF2‐multimer)^37^ and a later ColabFold version (group names ColabFold and colabfold_human),^38^ are a little way off the best performance possible, though each produces many high‐quality models (see below). Detailed descriptions of the best groups' methods are to be found elsewhere in this issue, but common themes are paying special attention to the MSA depth by collecting sequences from different databases, and sampling across models resulting from MSAs that differ in their depth, database origin, or sub‐clustering.

Third, despite the moderate performance of AF2 controls, most of the best groups used AF2 in one way or another: the best performing method that was entirely independent of AF2 was that of the BAKER group, 28th place, using RoseTTAFold2.^39^ Among those methods using AF2, there was an interesting variety of strategies although many groups placed significant emphasis on generating models based on diverse MSAs and selecting the best models from the resulting sets. There was also an interesting trend toward creatively combining AF2 predictions with alternative predictive methodologies. Thus, the UM‐TBM group^40^ used AF2 predictions to guide replica exchange Monte Carlo simulations within the I‐TASSER^41^ framework, while the Yang‐Server group^42^ selected from AF2 and trRosettaX2 results for its submissions. Finally, the pLM‐based methods (pink in Figure 1) are not currently competitive with MSA‐based methods like AF2: the best‐placed pLM group, ESM‐single‐sequence,^43^ is placed 74th among 118 evaluated participants.

Looking at the number of times each group produced the absolute best possible model (Figure S2) yields a slightly different perspective. PEZYFoldings^44^ remain the in first place by both CASP15 and GDT scores, the top server method UM‐TBM is in the second place by all scores, and Yang‐Server is placed third by GDT_HA. However, DFolding (fifth in the overall ranking in Figure 1) rises to third by composite CASP15 and GDT_TS scores, while ShanghaiTech (31st in the overall ranking) rises to fourth place in the CASP15 table and fifth in the GDT_TS ranking.

### 3.2 Group performance on different targets

**FIGURE 2:**
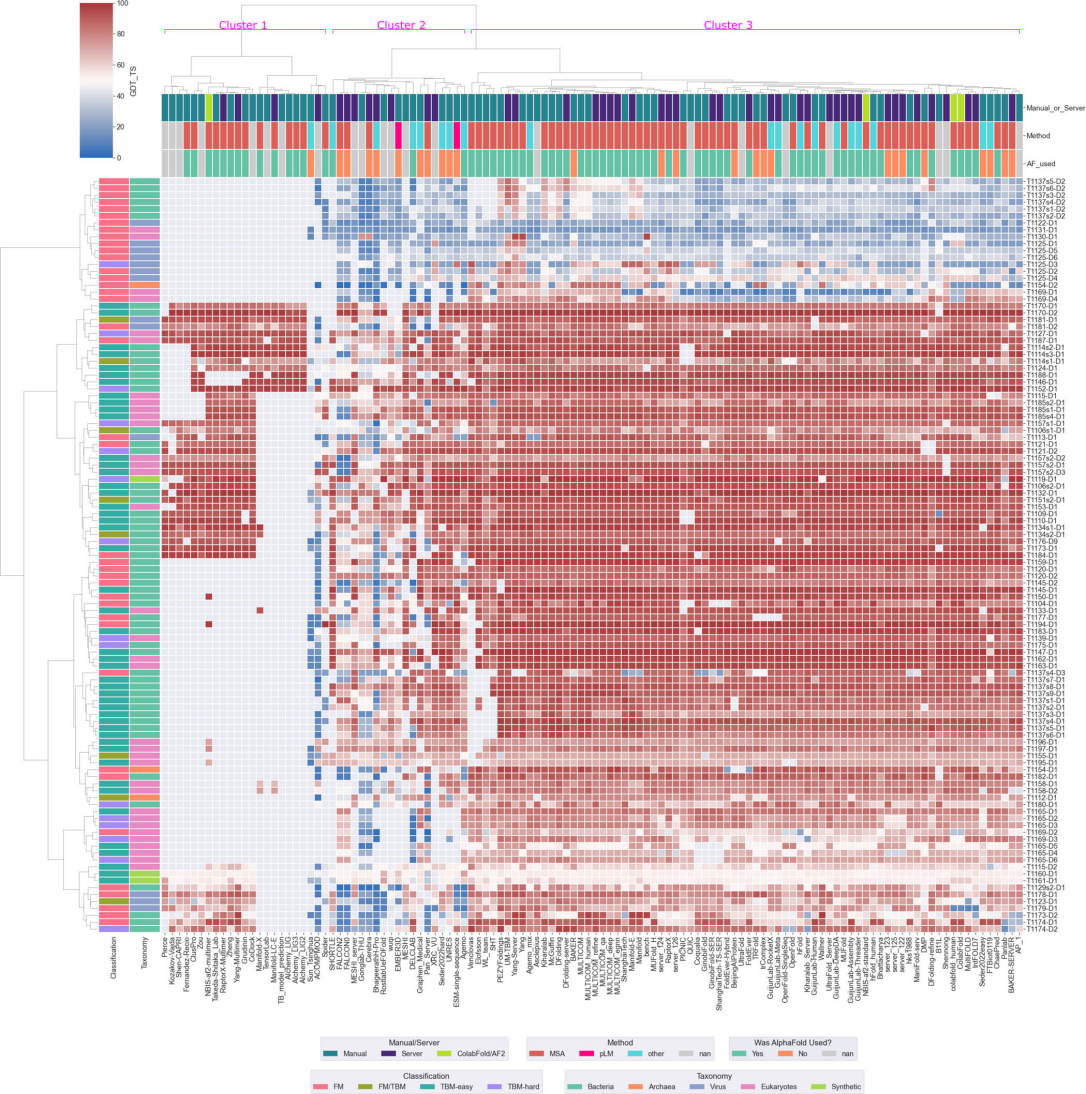
Ward's clustering applied to GDT_TS values (red good, blue bad, gray no submission) achieved by the 118 evaluated groups on the 109 Evaluation Units (EUs) that were considered. EUs are additionally annotated on the left with color codings relating to classification (TBM_easy, TBM_hard, FM/TBM, or FM) and taxonomy of the original target sequence (Bacteria, Archaea, Virus, Eukaryote, Synthetic). Groups are annotated on the top according to whether they were Server (indigo) or Human (blue), by broad category of method and according to if AF2 was used by a group. Note that the submitted Abstracts from some groups did not always allow confident inference of these aspects (gray labels). Three clusters of groups discussed in the text are indicated in magenta.

Figure 2 provides a two‐dimensional heat map of CASP15 groups and targets clustered based on the GDT_TS score. Groups to the left in Cluster 1 focused solely on multimeric targets. Cluster 2 contains a number of groups whose mixed blue and red coloring indicates variable performance: notably, these methods tended to not employ AF2 (or it was unclear from the submitted abstract whether that was the case). Cluster 3 includes a broad swathe of groups, many based one way or another on AF2, that produced largely good to excellent models over most of the target EUs. Table 1, for example, shows that selected methods (comprising the best‐performing MSA‐based methods, AF2 controls and the best pLM‐based method) produce model_1 predictions that have the correct topology (GDT_TS ≥ 45) in a large majority of cases. Remarkably, the best methods produce up to half of predictions with GDT_TS ≥ 90, a rough benchmark of differences between crystal forms of the same protein, and hence a reasonable ceiling on expected predictive performance. Again, two server groups that provide standardized versions of AF2: the NBIS‐af2‐standard, implementing a version of the original DeepMind AF2 protocol,^37^ and ColabFold^38^—perform somewhat worse than the top groups (Figure 2 and Table 1).

**TABLE 1:** Overall quality metrics for model_1 submissions by selected methods.

Method	GDT_TS ≥ 45 (total targets)	GDT_TS ≥ 90 (total targets)	Median GDT_TS
PEZYFoldings	101 (107) = 94.4%	53 (107) = 49.5%	89.65
UM‐TBM	105 (109) = 96.3%	46 (109) = 42.2%	87.36
Yang	105 (108) = 97.2%	51 (108) = 47.2%	89.26
ColabFold	93 (109) = 85.3%	42 (109) = 38.5%	86.67
NBIS‐af2‐standard	93 (109) = 85.3%	38 (109) = 34.9%	85.88
ESM‐single‐sequence	72 (93) = 77.4%	19 (93) = 20.4%	77.71

*Note*: The best‐performing MSA‐based methods, the AF2 controls and the best pLM‐based method were chosen for the comparison. The GDT_TS threshold of 45 corresponds to a prediction of the correct topology^8^; the 90 threshold is taken as the approximate difference between two crystal structures of the same protein.^7^

Looking next at the clustering by targets (vertical axis), there is a notable cluster of 18 at the top, for which average model quality was clearly lower, with most groups scoring GDT_TS < 50. Strikingly, 17 out of these 18 EUs were FM targets (the exception being T1125‐D3, classified as TBM‐hard), illustrating how the traditional four‐way classification of EUs by CASP,^11^ which is based on sequence and structural similarity of targets to PDB entries, still picks out targets that are more likely to prove difficult. However, it is important to note that another 22 FM targets were found in the main block of better‐predicted targets. Thus, FM targets are clearly predisposed to difficulties but may, depending on other characteristics (see later), still be well‐predicted. The other notable characteristic of the hard group of 18 was its richness in EUs derived from viral targets. Seven of the 18 are viral in origin, whereas the same is true only for 6 of the remaining 91 targets. This is a significant difference by Fisher's Exact test^45^ (*p* = .001; *p* < .05). Inspection of Figure 2 confirms that the best‐performing groups overall are those who also produced good models for the most challenging targets.

### 3.3 Target difficulty

#### 3.3.1 What makes a target difficult

Factors possibly explaining the greater difficulty of some targets, even to the best‐performing groups, were explored. The objective was to identify limitations of current methods and also to explain an apparent drop in overall performance by the best groups here compared to the best group at CASP14 (DeepMind with AF2). The latter issue is also explored in the introductory paper of this journal issue.^46^

**FIGURE 3:**
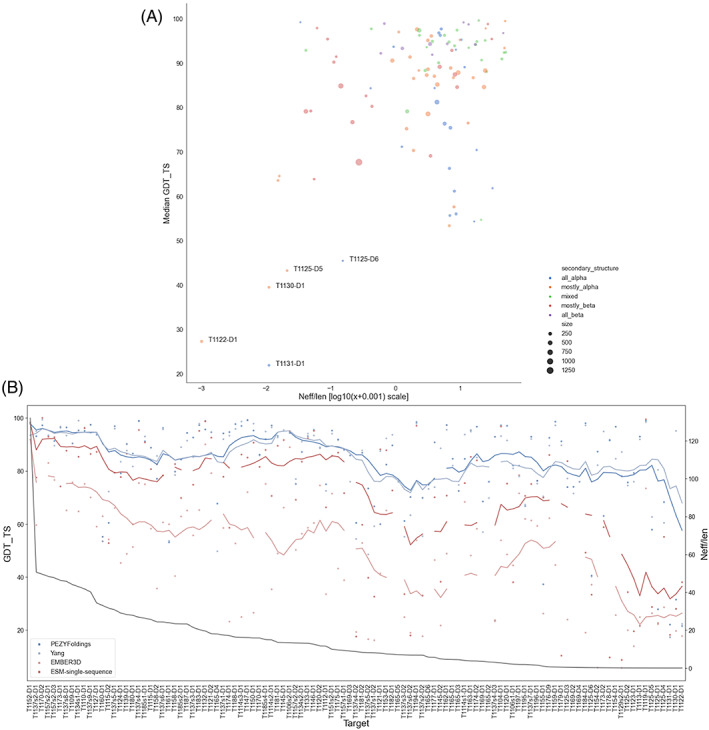
Analysis of performance versus Neff/length and other characteristics (A) Scatter plot of GDT_TS versus the log10 of target Neff/length + 0.001 for the top 10 groups. The scatter points are colored by secondary structure and the size of the points correspond to the size of the target. (B) Plot of GDT_TS versus target for the top performing MSA‐based methods (PEZYFoldings: Dark blue, Yang: Light blue) and the pLM methods (ESM‐single‐sequence: Dark red, EMBER3D: Light red). The lines represent a moving average for each method calculated across a 10 target window. The targets are ordered by Neff/length descending from left to right with Neff/len indicated on the right‐hand *y*‐axis.

Figure 3A illustrates a number of criteria that seem to link to target difficulty measured as median GDT_TS for the top 10 groups overall (Figure 3A). The most obvious characteristic of difficult targets is the availability of only a shallow MSA when homologues are collected from sequence databases. MSA depth here is measured as Neff^47^ normalized by length.^48^, ^49^ The hardest targets in Figure 3A all have very low Neff/length values: T1122, T1131 (the hardest target), and T1130 were all singletons in the principal public databases. The same trend persists in a graph calculated using models from all the groups (Figure S3). Evolutionary covariance information extracted from the MSA is used by methods such as AF2 to predict contacts and distances between residues, and is particularly key for AF2 for the derivation of an initial structure model.^37^ Where neither high‐quality covariance information nor a structural template (i.e., for FM targets) is available then even the best groups may struggle. This feature can be related to the abundance of virus‐derived targets in the most difficult group in Figure 2: as previously recognized, the rapid evolution of viral protein sequences can hamper the recognition and alignment of homologues in sequence databases.^8^

pLMs represent a new approach to structure prediction.^9^, ^10^, ^43^, ^50^ It has been asserted^9^, ^10^ that they may be less dependent on MSA‐derived information and hence potentially capable of comparatively better performance than MSA‐based methods on low Neff/length targets. Figure 3B, in which targets are ordered by their Neff/length, does not support this idea. The best pLM‐based methods are competitive with, though generally not quite as good as, MSA‐based methods for high Neff/length targets on the left. However, the performance deficit actually increases on the right as available MSAs become shallower.

Beyond low Neff, further analysis pinpoints other potential aggravating factors. For example, all of the five hardest targets are relatively small (Figure 3A), ranging in size from 66 to 234 residues, while no target larger than 300 residues achieved a median GDT_TS of less than 67. Similarly, the same five targets are either all‐α or mainly α by secondary structure composition and there seems to be a tendency for the more α‐rich targets to extend to lower median GDT_TS values among the results of the top 10 groups (Figure 3A). Finally, although numbers are small, it is interesting to note that four of the five hardest targets derive from crystal structures: the example of T1122 (below), though extreme, illuminates the particular complications that x‐ray crystallography may sometimes introduce.

#### 3.3.2 Examples of difficult targets

**FIGURE 4:**
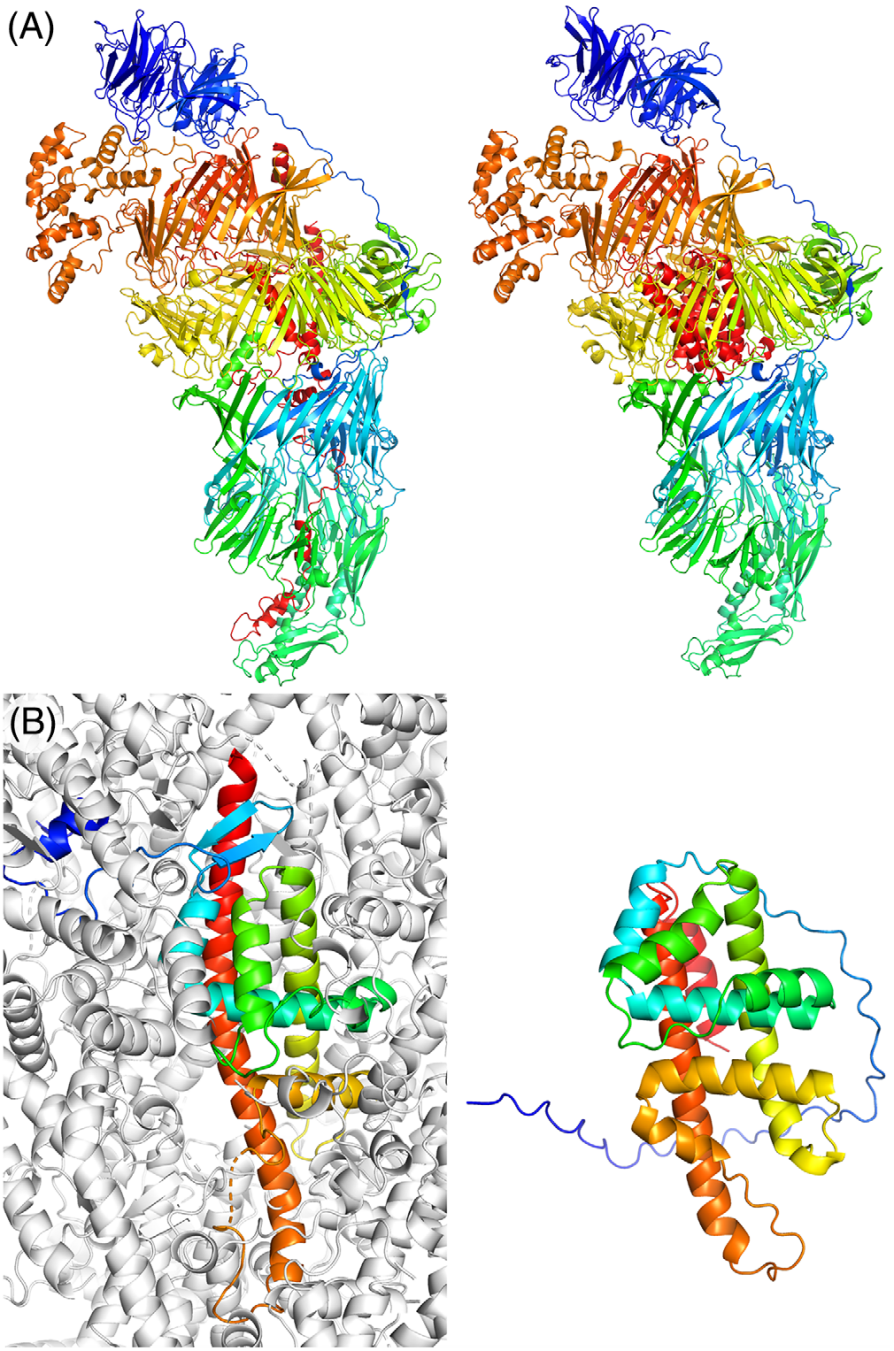
Examples of the best predictions produced for different targets. In each case, the experimental structure is shown on the left and the prediction on the right, each colored from blue to red from the N‐ to the C‐terminus. (A) T1169, at 2735 residues, is modeled with impressive overall accuracy by the Yang‐server group, with the exception of the C‐terminal 200 residues. (B) the T1122 crystal structure with only 25% solvent content has a tightly packed lattice producing abundant contacts between one subunit and its neighbors (symmetry mates are shown in gray), likely contributing to the poor quality of the best prediction (from the QUIC group).

Target T1169, mosquito salivary protein SGS1, was the longest single chain target yet seen at CASP and one of the longest single chains in the PDB. It is a hard modeling target that was divided into four assessment units, three FM, and one TBM‐hard.^11^ Despite this splitting, indicating that most groups struggled to predict the relative orientation of the individual units, some groups produced remarkably accurate predictions of most or even the entire structure. Figure 4A shows the top‐ranked prediction from the Yang‐server group, with a GDT_TS of 58 overall. With the exception of the C‐terminal 200 residues, the individual domains are accurately folded and packed against each other with truly impressive accuracy.

Targets T1130 and T1131 are aphid proteins which are, according to the submitting group, thought to be distantly homologous. They are both small (159 and 172 residues, respectively), largely α‐helical, monomeric, and were singletons in the main sequence databases at the time of CASP. A handful of good quality models of T1130 were submitted by groups who apparently discovered additional homologous sequences in the Supporting Information of a paper^51^ or in databases not searched by other groups.

Another small (241 residue) largely α‐helical monomeric target that proved difficult was T1122, a cranefly nudivirus protein with unusual properties related by the experimentalist submitters: derived from viral polyhedra, crystals of T1122 obtained in the 1950s have proved stable to this day, yet dissolving the crystal denatures the protein. Its crystal structure is also unusual in containing only 25% solvent leading to a densely packed protein array. Notably the N‐terminal 30 residues do not pack against the core of their own subunit, instead contacting four symmetry mates. Given its low Neff/length and the absence of this structural context as well as other contacting lattice mates numbering no fewer than 16, it is perhaps unsurprising that the best model_1, from the QUIC group, achieves a GDT_TS no better than 39 and only really authentically captures the packing of the core helices (Figure 4B).

### 3.4 Self‐assessment of results

CASP participants were asked to provide self‐assessment of their models at two levels: first, by supplying local per‐atom confidence values (expressed as pLDDT) in the B‐factor column of their submissions, and second, by ranking their submitted models (out of five allowed per target) in order of confidence. The local confidence estimates are particularly important since it is routine to trim off lower‐confidence regions for many applications of these models such as MR and structure searches of databases, while the model ranking is important as most user attention is likely to be focused on the top‐ranked prediction.

**FIGURE 5:**
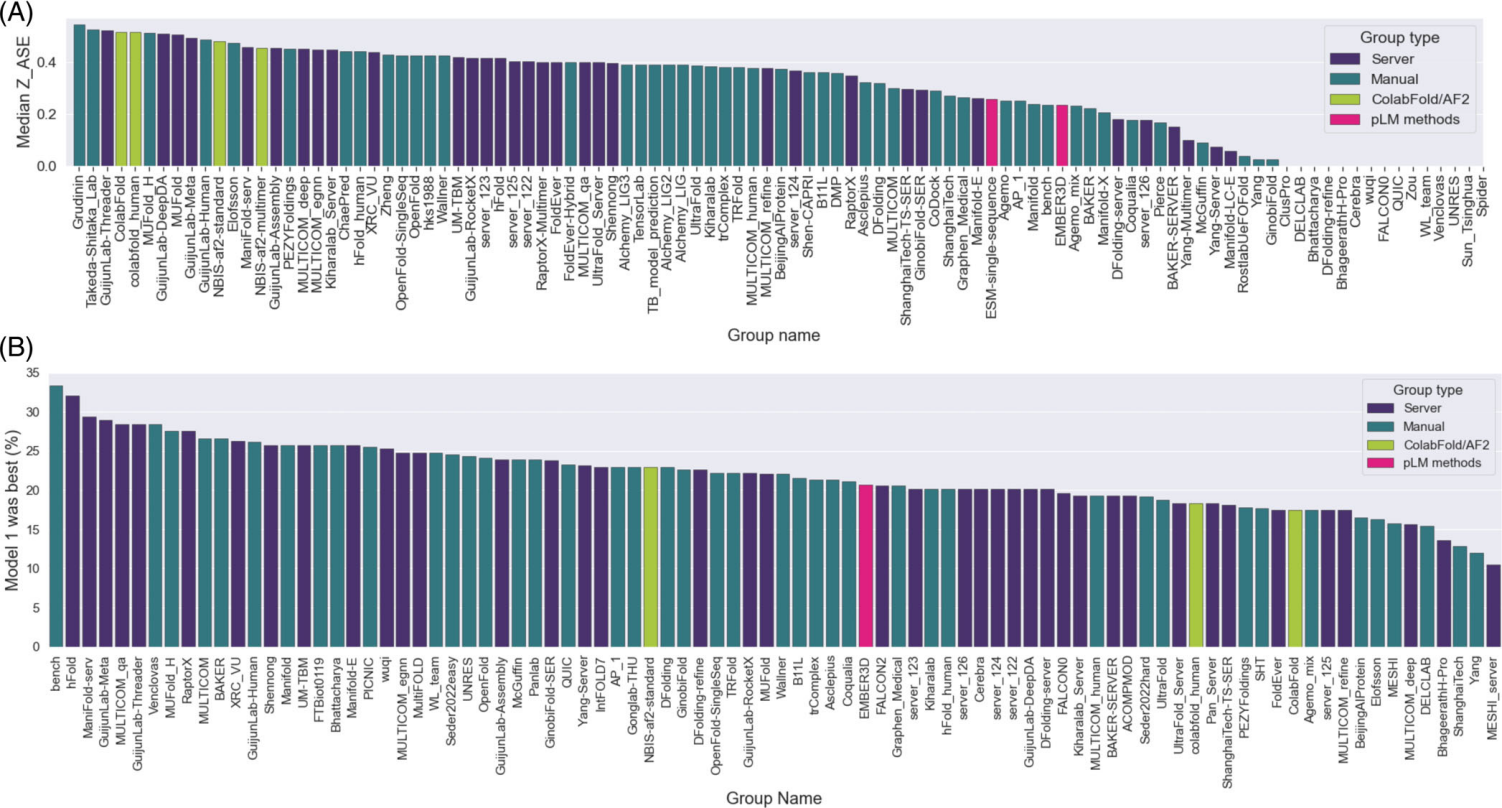
Group self‐assessment of results (A) groups ranked by median Z_ASE. (B) Groups ranked by how often Model 1 was the best model (expressed as a percentage). This ranking excludes groups which attempted less than half of the targets.

Detailed results of the local self‐assessment are provided elsewhere in this issue.^52^ Here, we provide a summary of the relative group performance according to their median ASE z‐scores across all submissions (Figure 5A). Notably, the confidence estimates produced by the control ColabFold and DeepMind AF2 entries are among the most accurate currently available, which should be seen as reassuring for users of the ColabFold pages^38^ and the AlphaFold Protein Structure DataBase,^53^ respectively. In comparison, the ESMFold quality estimates are a little less accurate, something users of the new ESM Metagenomic Atlas should bear in mind.^43^

Analyzing model ranking data, Figure 5B shows that only around two thirds of groups out‐perform the randomly expected 20% threshold of model_1 being the best submission (note that alternate conformation targets, where numbering of models is irrelevant for the ranking purposes, were not considered here). For example, the overall winning group PEZYFoldings and the ColabFold group are each a little below 20%. However, AF2‐based methods such as ColabFold often produce several predictions that are very similar: it is clearly a greater challenge to select the best in this situation than to spot the best from among five very divergent predictions.

### 3.5 Factors affecting local accuracy

**FIGURE 6:**
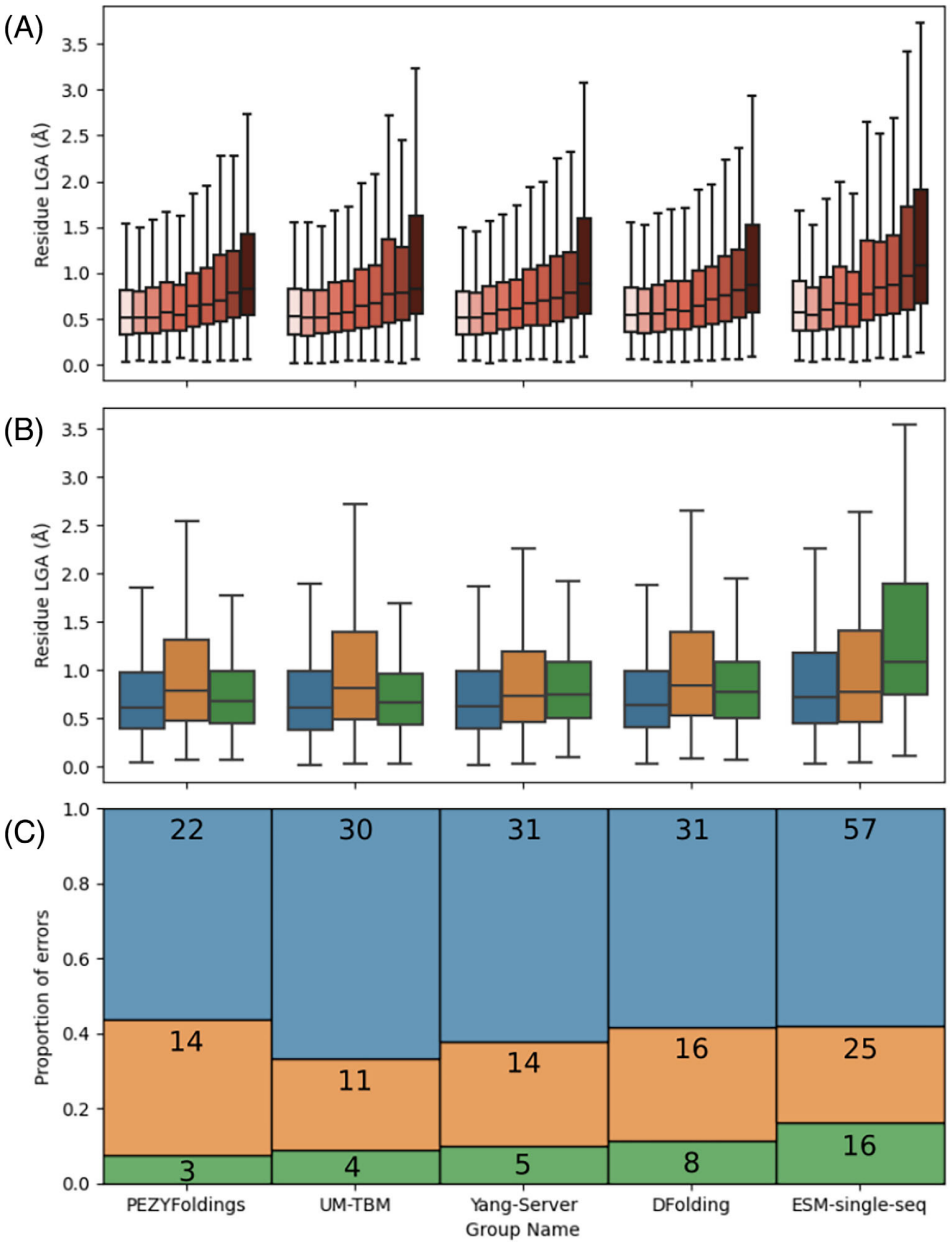
Factors affecting local accuracy analyzed using the results of selected MSA‐ and pLM‐based approaches. Only high‐quality (GDT_TS > 80) model_1 submissions are considered. (A) Residue LGA error tends to correlate with normalized B‐factor: for each method, residue LGA error increases from low (light color) to high (dark color) bins of normalized B‐factors. (B) Distribution of LGA error values across residues observed neighboring a crystal lattice interface (orange), a chain interface (green), or neither (blue). (C) Error regions (defined in Materials and Methods) are classified according to their presence at a crystal lattice interface (orange), at a chain interface (green) or neither (blue).

Where a portion of a target structure does not necessarily capture the only accessible conformation, then it is unreasonable to expect the prediction to necessarily resemble the target. Flexible surface loops are likely to be difficult to predict since there can be multiple conformations differing little energetically. Some loops will be tethered by interactions in a crystal structure, but others will retain flexibility in the context of the crystal lattice resulting in locally smeared out electron density and higher local B‐factors. For four selected Deep Learning methods and the pLM method, ESM‐single‐sequence, the relationship between experimental B‐factors and residue LGA error was studied (Figure 6A). Only higher‐quality (GDT_TS >80) model_1 submissions were considered (numbering from 30 in the case of ESM‐single‐seq to 46 for PEZYFoldings and 47 in the cases of UM‐TBM, DFolding, and Yang‐Server). Binning residues in these predictions by normalized B‐factors (see Materials and Methods) and assessing the residue LGA error range in each bin reveals a strong relationship. For all methods, mean bin error increases with increasing normalized B‐factor (Figure 6A).

It is understood that the formation of the crystal lattice can lead to local distortion of residues away from the most energetically stable conformations. Indeed, it has been argued previously that where a high‐quality structure prediction differs from the target at a crystal lattice, it should not automatically be inferred that the crystal structure is correct and the structure prediction in error.^24^ Another way to view the situation is that the structure prediction program lacks the 3D context—the neighboring crystal symmetry mates—that would help it accurately predict crystal lattice structures. A similar logic can be applied to interfaces between chains: lack of structural context means they may well prove harder to predict where only a single chain is being modeled. These questions were explored here first by looking at mean local error among interface residues compared to others; and second by defining error regions (see Materials and Methods) and checking whether there was evidence of their over‐representation at crystal lattice or chain interfaces. Analysis of domain interfaces was also attempted (not shown) but the number of such residues was too small to allow meaningful analysis.

Considering first the local LGA errors (Figure 6B), residues at crystal lattice interfaces have significantly higher errors than non‐interface residues for the results of all five groups considered: two sample *t*‐test results gave *p*‐values running from 2.73 × 10^−13^ to 0.04. Although the difference was often less pronounced, localization at a chain interface also resulted in significantly higher local errors in the results of all methods except PEZYfoldings. Similar results were obtained when considering error regions (see Materials and Methods) of at least three residues (Figure 6C). As expected, the pLM method ESM‐single‐sequence that is less accurate overall had larger numbers of such regions than the MSA‐based methods. However, across all methods, a consistent proportion of around 40% of error regions are found at crystal lattice or chain interfaces, with the former always significantly outnumbering the latter. Taken together, these results show that even the best predictive methods can still struggle with interface regions, especially crystal lattice contacts. However, the question remains, remembering the local forces exerted on proteins as they crystallize, as to whether crystal lattice “mispredictions” should be regarded as errors: the model region may represent an alternative correct, biologically accessible conformation or even, if the lattice interface is distorted, the single biologically relevant conformation. A similar situation applies for high B‐factor flexible loops where model and target, though different, may be equally valid snapshots from a biological ensemble. A continued recognition at CASP of these mitigating factors around some “mispredictions” is important to define the scope for future improvement of the current state of the art.

### 3.6 Side chain accuracy

As explained earlier, side chain accuracy is one component of the overall composite score but, as global main chain quality has improved, particularly post‐AF2, improving side chain placement is seen as an important area for future development. Assessing reasonable expectations for optimal side chain placement is complicated by the fact that surface‐located side chains will often have multiple, significantly occupied conformations. These may or may not be resolved experimentally, depending largely on the resolution of the data available.

**FIGURE 7:**
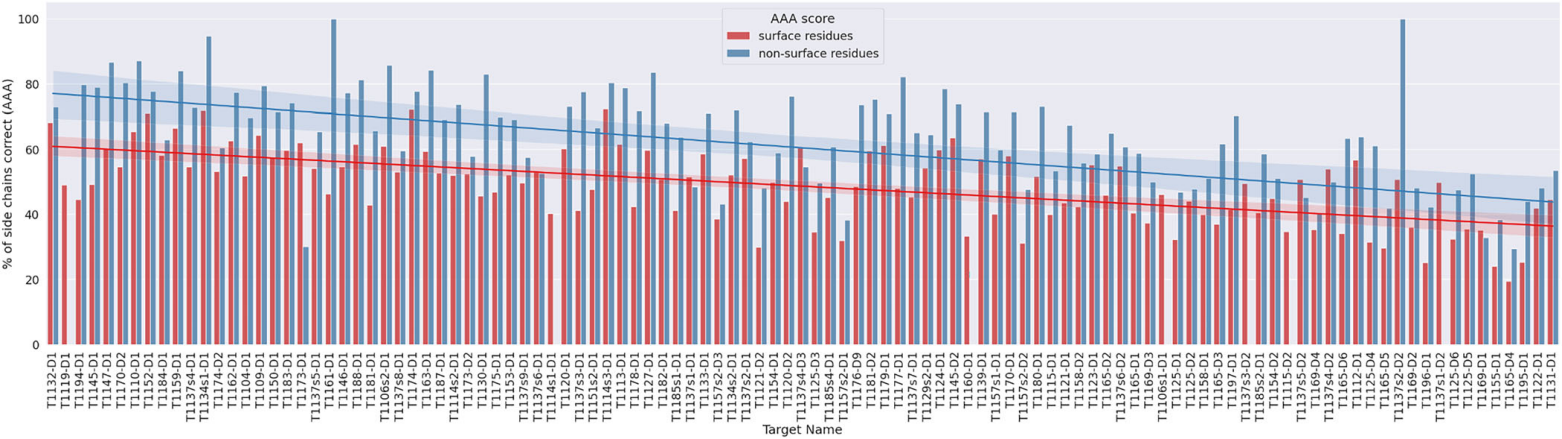
Per‐target comparison of the mean SCWRL4 AAA sidechain score for surface residues (red) and non‐surface residues (blue) for the model with the highest GDT_HA for each target. Residues were defined as surface residues if their solvent accessibility was ≥20% as given by the Shrake–Rupley algorithm. A line of best fit is shown for both the surface and the non‐surface residues in corresponding colors. The targets are ordered in descending order by the GDT_HA value of the top model.

To assess if surface side chains were more difficult to model than non‐surface side chains, the SCWRL4 AAA sidechain scores^21^ were calculated for different types of the side chains (see Materials and Methods; Figure 7). Seven targets were classified as all‐surface (T1106s1‐D1, T1114s1‐D1, T1115‐D2, T1119‐D1, T1137s1‐D2, T1137s3‐D2, and T1160‐D1). For the remainder, the non‐surface sidechain score was higher than the surface score in all but seven targets (T1173‐D1, T1137s1‐D1, T1137s4‐D2, T1137s4‐D3, T1137s5‐D2, T1137s6‐D1, and T1169‐D1) out of the 109 targets. Remarkably, for two targets (T1161‐D1; TBM‐easy, and especially T1137s2‐D2; FM) all non‐surface side chains were correct. Figure 7 shows that side chain accuracy tends to decline, left to right, as the accuracy of the best model decreases. Naturally, the proportions of surface and non‐surface residues vary across the set of targets: the median ratio of surface: non‐surface residues was 2.2:1.

**FIGURE 8:**
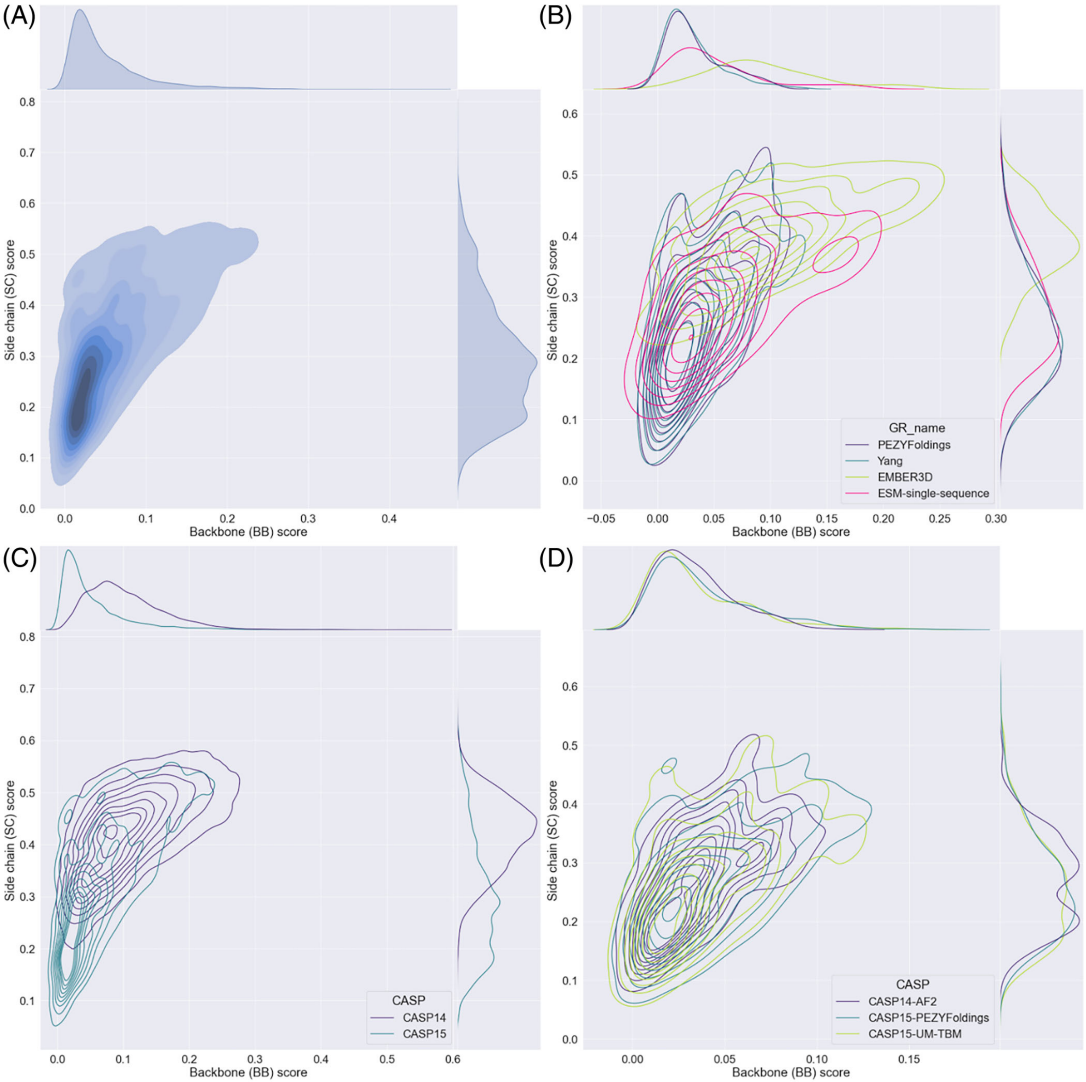
Contour plots illustrating the relationship between backbone and side chain dihedral scores (see Materials and Methods), calculated across whole models. Each contour plot is supplemented by a plot on the right illustrating the distribution of side chain scores, and one above showing the main chain score distribution. (A) Shows all groups, all models in CASP15 illustrating how a good (low) backbone (BB) score is necessary but not sufficient for a good (low) side chain (SC) score. (B) Shows a comparison between the models produced by two of the best MSA‐based methods—PEZYFoldings and Yang, and two pLM methods—EMBER3D and ESM‐single‐sequence. (C) Shows a comparison between all groups, all models in CASP14 (indigo) and CASP15 (teal). (D) Shows a comparison between AF2 in CASP14 and the two top performing methods in CASP15 (PEZYFoldings and UM‐TBM).

The side chain (SC) and backbone (BB) dihedral‐based accuracy measures were plotted against each other to assess their relationship (Figure 8A). In each case, low values indicate high accuracy. The result shows a sharp dependency—as expected, high accuracy backbone structure is required before highly accurate side chain placement becomes possible.^37^ Nevertheless, even with near‐ideal backbones, side chain scores never approach 0 (presumably due to the alternate conformation issue mentioned above). Equally striking, high‐quality backbone structures do not guarantee successful side chain placement, illustrating how these are connected but still distinct challenges for predictors.

Figure 8B shows the individual performance of the two best groups using each of the MSA‐based and pLM methods. Notably, the pLM methods, especially EMBER3D, lag behind the MSA‐based methods in terms of side chain prediction: for models of a given low backbone score, reflective of high quality fold prediction, pLM methods give poorer side chain placement. Figure 8C,D illustrates the progress from CASP14 to CASP15 in this aspect of modeling. The dramatic progress seen in Figure 8C is illustrative of the transformation brought about by AF2: the much better BB scores seen for the largely AF2‐based methods at CASP15 allow, in turn, much better SC scores. Perhaps less predictably, the top two methods at CASP15, PEZYFoldings, and UM‐TBM, do seem to out‐perform the AF2 entrant at CASP14 in terms of side chain scores (Figure 8D). Notably, the abstract from the UM‐TBM team commented that its refinement element was designed specifically to improve side chain accuracy. The distribution of SC scores shows that it does slightly outperform PEZYFolding in the proportion of low SC score models.

### 3.7 Molecular replacement

Since MR is an important downstream application of protein structure modeling,^20^, ^54^ submissions were also assessed directly for their suitability to serve as MR search models. As mentioned above, the reLLG^20^ provides a coordinates‐only metric for this purpose, and was newly included in the overall CASP15 score. However, where diffraction data were available (in 17 cases—see Table S1) they were used for a more direct assessment of the CASP15 submissions. This was done first by calculating log‐likelihood‐gain values (LLGs) for models ideally placed by superposition on the target crystal structure and refined; and second by carrying out full MR using CASP submissions as search models.

#### 3.7.1 Assessing the models' potential for success in MR

**FIGURE 9:**
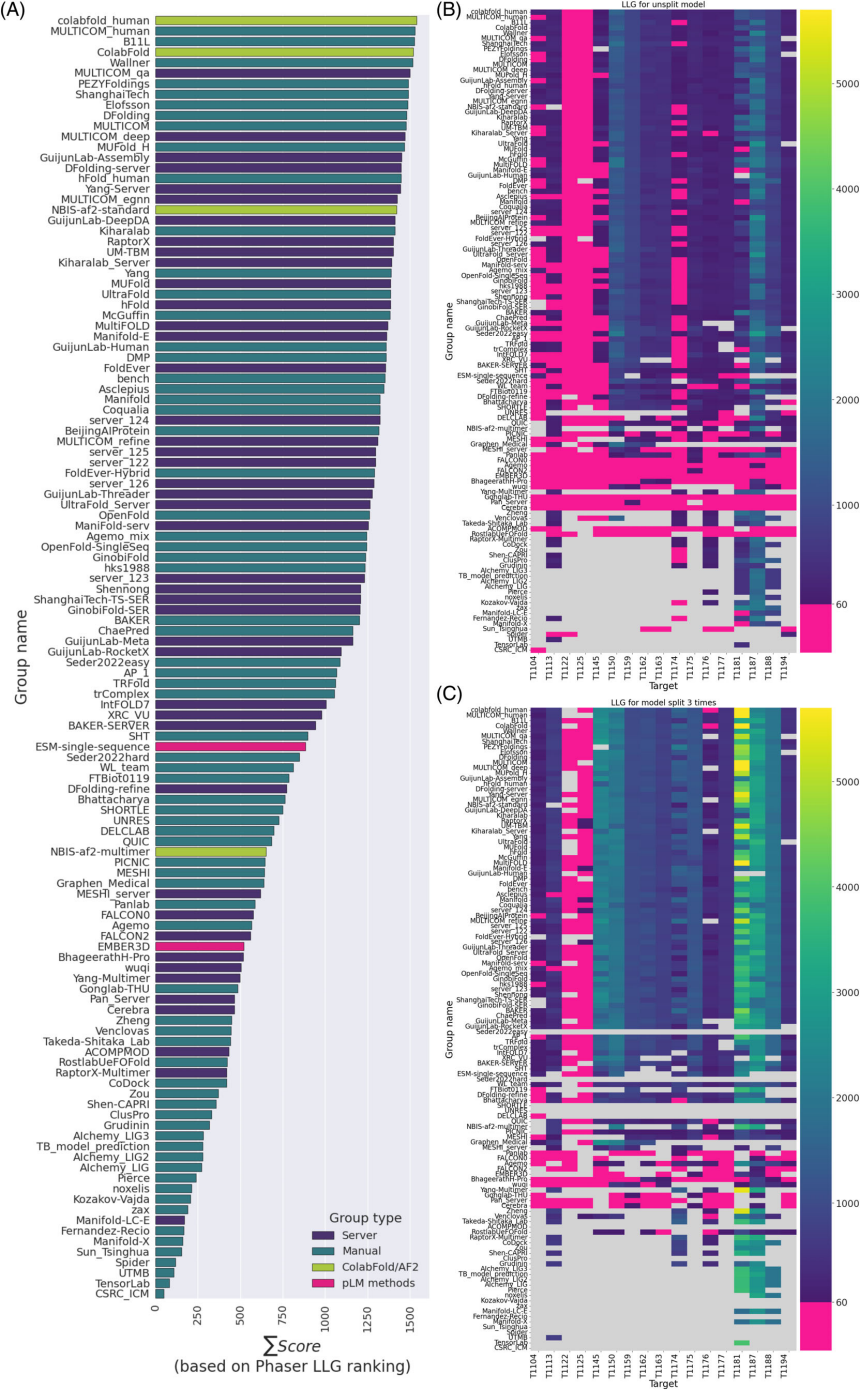
(A) Groups ranked by the cumulative LLG‐derived ranking score described in Materials and Methods. (B,C) A comparison between the LLG scores for an ideally placed model (B) before splitting and (C) after splitting three times using the Birch algorithm in Slice'N'Dice. Pink indicates LLG scores below 60, the success threshold in MR. The blue to yellow gradient (see the coloring map next to the graph) depicts the LLG scores greater than 60 with yellow indicating the largest LLG values. Gray denotes instances where groups did not submit models for a target or where Phaser failed to produce a solution. Groups are ordered the same in all three panels.

Submissions were processed to remove low confidence (pLDDT < 70) regions, fit onto the target structure with Gesamt,^28^ and finally refined with Phaser^29^ (see Materials and Methods for details). In this way, a LLG was obtained for model_1 submissions for each target and each group, allowing ranking of groups for each target. Converting the ranking into a score (see Materials and Methods), allowed for a comparison of groups across all targets (Figure 9A). The best scoring group was Colabfold_human and, in general, the best groups were those using AF2. Search models from pLM methods scored less well but proved to be good enough to exceed the LLG = 60 threshold for many of the targets, indicating their potential utility in solving the MR problem.

In MR, it is common to split a potential search model and place the resulting domains separately. This addresses the possibility that the domain orientation in the target may be different to that in the available search model(s), whether as a result of inaccurate structure prediction or simply because of different biologically relevant inter‐domain orientations. For each target, except T1122 and T1125, several of the search models created from the unsplit CASP submission already scored well enough (LLG > 60) to indicate potential success in MR (Figure 9B). However, when the models were split into three pieces, LLG scores improved (Figure 9C), most notably for larger targets such as T1145 (636 residues), T1174 (339 residues), and T1181 (689 residues). In addition, some of the split models showed potential for structure solution of T1125, scoring an LLG better than 60.

#### 3.7.2 Full MR

**FIGURE 10:**
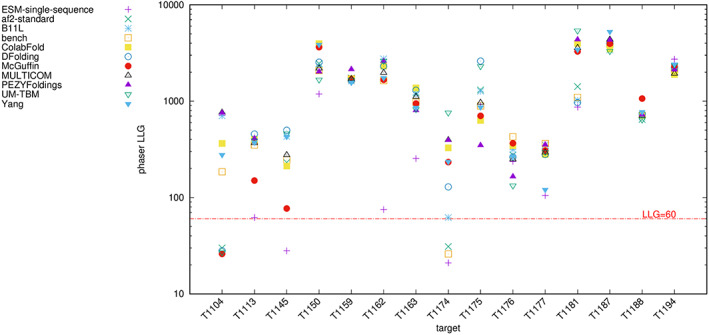
LLG values from full MR tests for unsplit model_1 predictions for 11 selected groups, modified to remove residues with pLDDT < 70, and placed by Phaser. T1122 and T11225 are not shown since no search model produced a solution.

Even a search model with a high LLG after ideal placement may not succeed in full MR because of issues such as packing clashes. In order to assess real‐world performance, Model 1 from selected best ranking groups in the alignment tests, including the best scoring pLM‐based group ESM‐single‐sequence, was used for full MR. The unsplit models, subjected to pLDDT‐to‐B‐factor conversion and removal of residues scoring pLDDT < 70, were used. Figure 10 shows the results of these tests, displaying the LLG from Phaser for each model and each target. No group produced a successful search for targets T1122 and T1125. For the remaining targets, many of the groups produced models that could be successfully placed in the MR search. The ESM‐single‐sequence models were the least successful, although they were sufficient for use as search models in several cases.

#### 3.7.3 MR using ESM‐single‐sequence model for T1145

**FIGURE 11:**
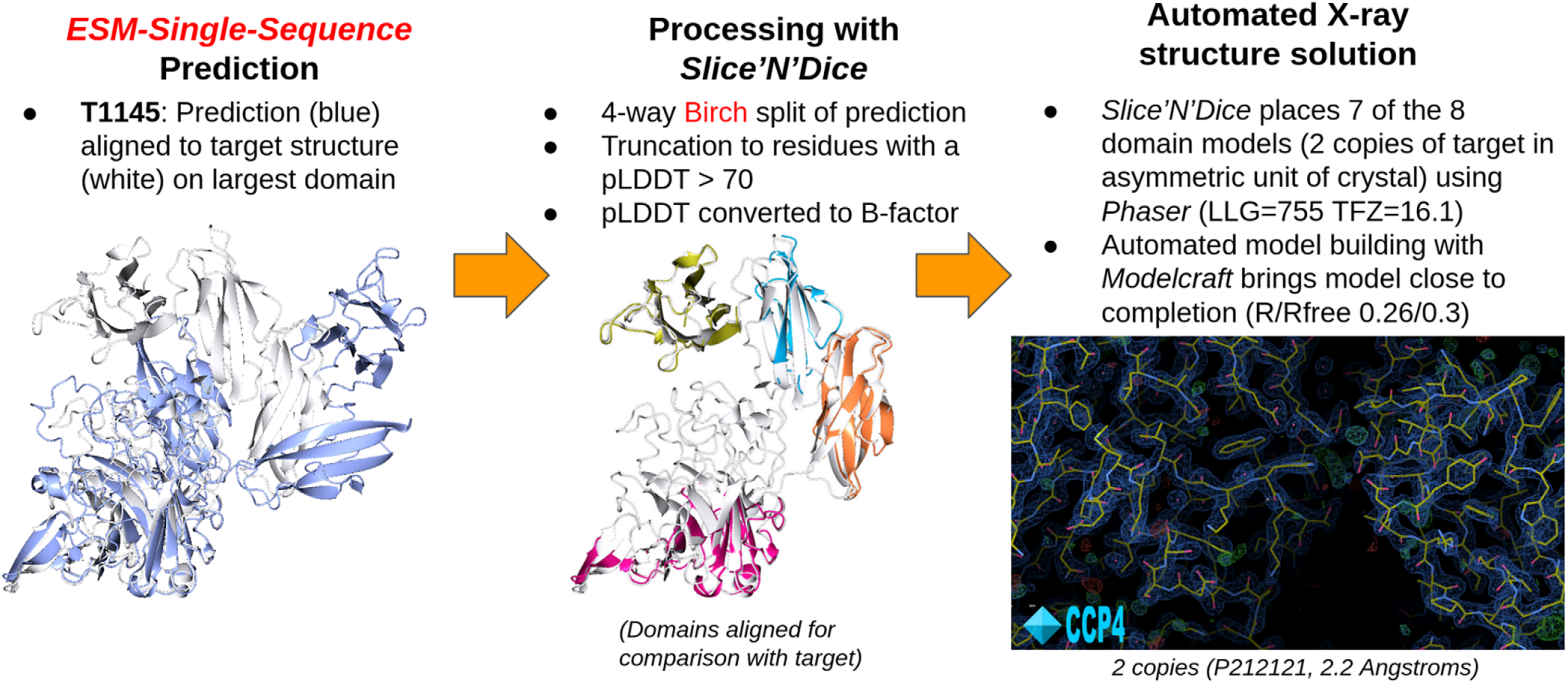
Using Slice'N'Dice to automatically split the ESM‐single‐sequence predicted model_1 for T1145 into four domains (different colors; also retaining only residues with pLDDT > 70) and perform MR using Phaser. Phaser places seven of the eight domain models and further completion is achieved using the Modelcraft model building application.

One of the targets where the ESM‐single‐sequence model failed to produce an MR solution was T1145. We examined this case in more detail to test whether a predicted model, which differs greatly in its overall conformation from the crystallized form, could be successfully used in a split form to produce a correct MR solution. Figure S4 shows the results of the aligned model test for T1145 for Model 1 from all groups. It shows that, when the ESM‐single‐Sequence model is split into 2, 3, or 4 pieces using the SnD application, the resulting LLGs from Phaser strongly suggest the possibility of successful MR. Testing this hypothesis by full MR, we found that the optimal splitting was divide the predicted model into four domains (Figure 11). Target T1145 derives from a crystal structure containing two copies of the target in the asymmetric unit cell. Phaser successfully positioned seven of the eight domains producing phases and allowing calculation of an initial electron density map. The map was of sufficiently good quality for the model building application Modelcraft^34^ to successfully build most of the two copies of the target structure. Restrained refinement using Refmac5 achieved an Rfactor/Rfree of 0.26/0.3 after model building, showing good agreement between the refined structure and the observed reflection data. These are typical values for what can be achieved in the automatic model building of a macromolecular crystal structure. Further completion of the structure usually requires manual effort through a graphical interface.

### 3.8 Function prediction

The interpretation and prediction of the functions of proteins based on their modeling is of major importance.^55^ Some function predictions rely on global properties, such as inference of nucleic acid binding capability based on electrostatic properties,^35^ and are therefore tolerant of some error. Other methods are acutely dependent on the accurate capturing of fine details such as the local conformations of specific residues responsible for ligand recognition. With this in mind, targets were selected based on their interpretability by structure‐based methods. Target selection was based on information given to the CASP predictors in combination with analysis and literature review of the targets. Four enzymes T1146, T1110, T1127, and T1188 with catalytic dyads or triads were selected as well as one DNA‐binding protein (T1151).

**FIGURE 12:**
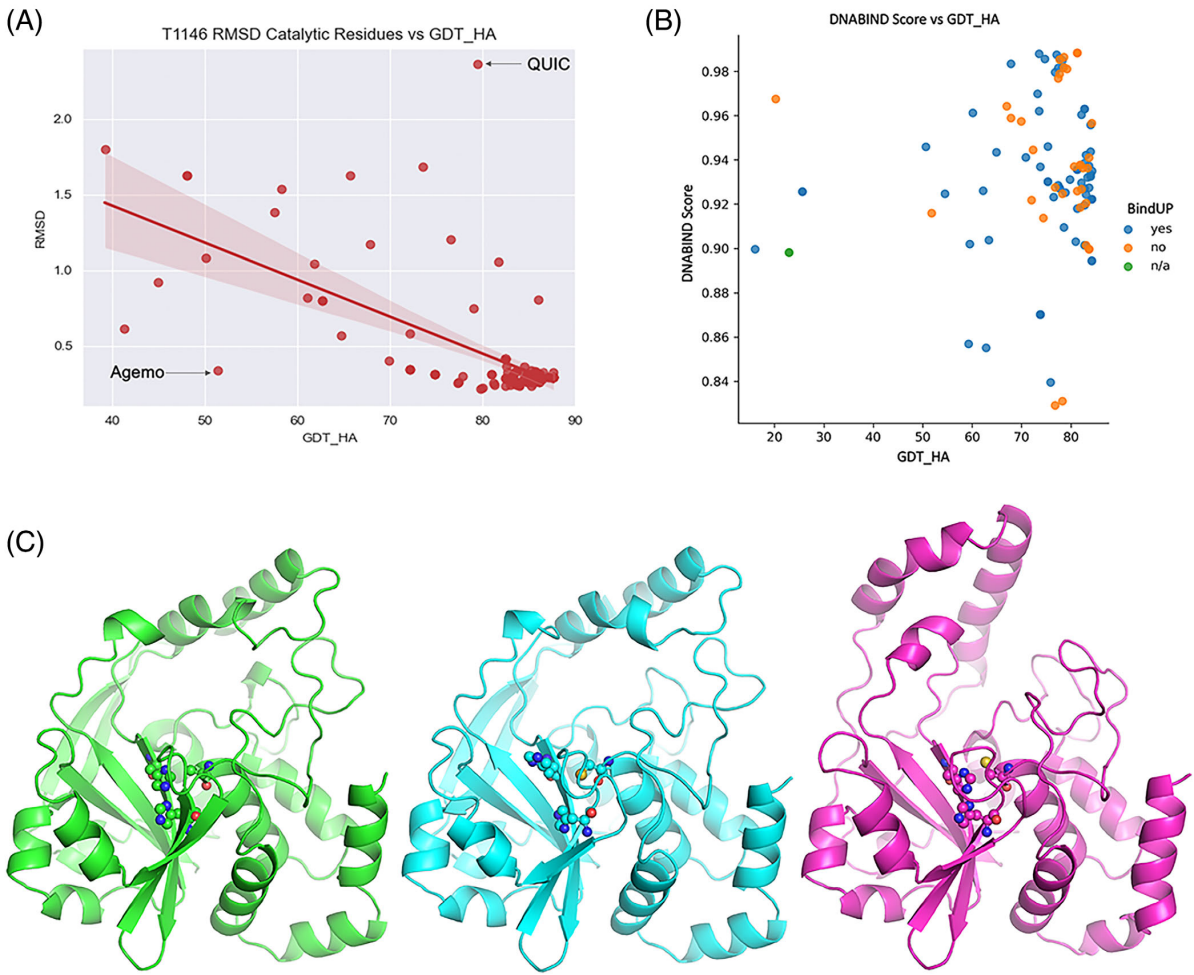
Function prediction based on submitted models. (A) Global accuracy and the accuracy of functional features are only weakly correlated, as exemplified here by the RMSD on catalytic residues versus GDT_HA for T1146. (B) DNABIND probability score against Global accuracy. The coloring of the data points indicates BindUP results: blue—positive, orange—negative, green—could not be processed. (C) The T1146 catalytic triad (sticks) and overall fold (cartoon) in the experimental structure (green) and two outliers; cyan—highly accurate fold, wrong conformation of one catalytic His (Model 1 for the QUIC group); magenta—fold prediction less accurate, but catalytic site well‐modeled (Model 1 for the Agemo group).

The ability to detect the catalytic sites by matching 3D structural motifs depends on their accurate local modeling. To assess their predictability given a certain accuracy of global modeling, the value deriving from all atom fitting of the catalytic residues of the active sites to templates was plotted against measures of global fold quality and side chain metrics. The global accuracy metric (GDT_HA) and side chain metrics (data not shown) are not strongly correlated with the RMSD of the catalytic residues, and there are outliers. Figure 12A shows this trend for the T1146 target; other targets are shown in Figure S5. For example, Model 1 from the QUIC group for the T1146 target has a high GDT_HA (79.4) but the RMSD for the catalytic triad residues is relatively high (2.36 Å). Inspection of this model reveals that the protein has a very accurate fold but one of the catalytic residues (His255) is in the wrong conformation (Figure 12C). Conversely, Model 1 from the Agemo group has a low GDT_HA (51.3) but the RMSD (0.34 Å) for the catalytic triad residues is relatively low. Inspection of the model demonstrated that the overall fold is poorly modeled in respect of its relative domain orientation, yet the catalytic triad residues are correctly placed relative to each other (Figure 12C).

For T1151, all models score above the DNABIND default threshold (0.531) and are predicted as DNA‐binding. However not all models are predicted as potentially DNA‐binding by BindUP (Figure 12B). In this regard, overall model quality seems to have little impact on DNA‐binding prediction; there is no general trend between GDT_HA and the DNABIND probability score, nor is there a tendency for better models to predict as DNA‐binding by BindUP. These findings are in line with the known error tolerance of the DNABIND method.^35^

## 4 CONCLUSIONS

The first conclusion of single chain assessment at CASP15 must be that AF2 remains the dominant presence in the field: the best‐ranking group that did not use it in one form or another was the BAKER group in 28th position in the overall rankings. Set against this, however, must be placed the observation that groups used AF2 in diverse ways including, intriguingly, in combination with previous generations of structure prediction software. Thus, the UM‐TBM group hybridized AF2 predictions with the I‐TASSER framework^40^ and the Yang‐Server group sampled from both AF2 and trRosettaX2.^42^ Nevertheless, the overall winner PEZYFoldings used AF2 in relatively orthodox fashion for construction of models: a novel, additional post‐prediction refinement step based on a fine‐tuned AF2 turned out, on closer examination, to only modestly improve their already excellent models.^44^

At CASP14, AF2 was far ahead of other groups.^7^, ^8^ This time, a large number of groups, mostly using AF2 results in one fashion or another, produced excellent models for most targets (Figure 2). What differentiated the best groups was their ability to produce good models for the most difficult set of EUs. Compared to previous CASPs, there were more FM targets whose absence of templates obviously provides a first element of difficulty. Accordingly, further analysis shows that the most obvious characteristic shared by the hardest targets was a lack of detectable homologous sequences, leading to shallow MSAs and weak or even absent covariance information. This likely relates to the over‐representation of fast‐evolving viral sequences in the set of hardest targets. Inferred distance information from covariance analysis is known to be crucial for the initial model estimation by AF2 where templates are not available.^37^ The advantages gleaned by the best‐performing groups seem to partly derive from an ability to scrape hidden sequence information from sources not necessarily included in the main databases. In an ideal world, a single, unified, and comprehensive database would be available to all groups so that performance disparities could be related more directly to differences in predictive methods. Aside from the number of homologues, there are hints that harder targets may be more likely to be smaller and predominantly α‐helical in secondary structure. This tentative observation is in complete contrast to results in the time of fragment assembly ab initio methods, for example,^56^ when these targets were generally the most favorable and is worthy of further study on a larger scale.

Even models that are globally accurate can contain regions that match the target less well. These may result straightforwardly from poorer predictive performance but other explanations are possible, and analysis supports the relevance of two other factors. First, proteins are naturally flexible and such motions can occur in the particles analyzed by cryo‐electron microscopy and even in a protein crystal. Such mobile regions have a variety of accessible conformations meaning that a failure of the modeling to capture the same local structure seen in the target is not necessarily indicative of erroneous modeling. In‐crystal movement can result in elevated B‐factors for the affected part(s) or even, in extreme cases, the complete absence of electron density. The clear trend found between higher B‐factors and higher local errors (Figure 6A) suggests that in some cases the difference between prediction and target cannot necessarily be straightforwardly inferred as wrong, potentially instead being a different valid structure.

A second feature affecting interpretation of local accuracy is positioning at an interface, either at a crystal lattice interface or with a different chain in an oligomeric structure (Figure 6B). Since lattice formation can distort lattice contacts away from conformations accessible in solution, a deviation of a prediction from target at the interface may be an alternative valid structure, or even conceivably more correct than a potentially distorted conformation in the target.^24^ Inter‐chain interface regions are likely to be more difficult to predict than other parts of the target because their local conformation may depend on 3D structural context that is absent during the modeling process.

While analysis has focused on difficulties and room for future improvement, it should be remembered that the overall picture is one in which many groups produce remarkably good models for most targets (Figure 2, Table 1). Furthermore, as also noted elsewhere,^20^, ^54^ outputs from readily available methods like ColabFold^38^ and ESMFold^43^ (or deposits in databases generated by similar protocols^43^, ^53^) can solve most crystal structures by MR (Figures 10 and 11); are similarly valuable in solution of structures determined by cryo‐electron microscopy as starting models for density‐guided refinement into experimental data^57^; and often allow functional annotation by accurately capturing key local features (Figure 12). In this respect, much credit goes to DeepMind for making AF2 Open Access and thereby democratizing state‐of‐the art‐modeling and reinvigorating whole areas of research. Notably, since human groups have typically out‐performed servers at previous competitions, and recalling that the stand‐out CASP14 winner DeepMind competed as a human group, the strong representation of automated servers among the very best groups at CASP15 is a welcome development (Figure 1, Figures S1 and S2). In summary, while further methods development will proceed apace, addressing issues such as side chain accuracy^58^ and targets with few homologues, colleagues across biology already have immensely powerful tools whose applications will only continue to expand.

## AUTHOR CONTRIBUTIONS

**Adam J. Simpkin:** Conceptualization; methodology; software; data curation; writing – review and editing; visualization; investigation; writing – original draft. **Shahram Mesdaghi:** Methodology; data curation; investigation; visualization; writing – review and editing; writing – original draft. **Filomeno Sánchez Rodríguez:** Conceptualization; methodology; software; data curation; investigation; visualization; writing – original draft; writing – review and editing. **Luc Elliott:** Investigation; visualization; software; data curation. **David L. Murphy:** Investigation; visualization. **Andriy Kryshtafovych:** Conceptualization; methodology; visualization; data curation; funding acquisition; writing – review and editing. **Ronan M. Keegan:** Conceptualization; methodology; software; data curation; investigation; visualization; writing – original draft; writing – review and editing. **Daniel J. Rigden:** Conceptualization; methodology; data curation; investigation; visualization; writing – original draft; writing – review and editing; project administration; supervision; funding acquisition.

## Supporting information


**DATA S1:** Supporting Information.

## Data Availability

The data that support the findings of this study are available from the corresponding author upon reasonable request.
