# Supplementary material for: Tertiary structure assessment at CASP15
Source: Proteins. 2023 Sep 25;91(12):1616–35. doi: 10.1002/prot.26593 (PMC10792517; doi:10.1002/prot.26593)
Supplement: Supplementary file 1 — DATA S1: Supporting Information. [file PROT-91-1616-s001.docx]

**Tertiary structure assessment at CASP15**

**Supplementary Material.**

Adam J. Simpkin^1^, Shahram Mesdaghi^1^, Filomeno Sánchez Rodríguez^1,2,3^, Luc Elliott^1^, David L. Murphy^1^, Andriy Kryshtafovych^4^, Ronan M. Keegan^5^, Daniel J. Rigden^1^ *

^1^ Institute of Structural, Molecular and Integrative Biology, University of Liverpool, Liverpool L69 7ZB, England

^2^ Life Science, Diamond Light Source, Harwell Science and Innovation Campus, Didcot, Oxfordshire OX11 0DE, England

^3^ York Structural Biology Laboratory, Department of Chemistry, University of York, Heslington, York, England

^4^ Genome Center, University of California, Davis, California

^5^ UKRI-STFC, Rutherford Appleton Laboratory, Research Complex at Harwell, Didcot OX11 0FA, England

*Correspondence e-mail: [drigden@liverpool.ac.uk](mailto:drigden@liverpool.ac.uk)

###
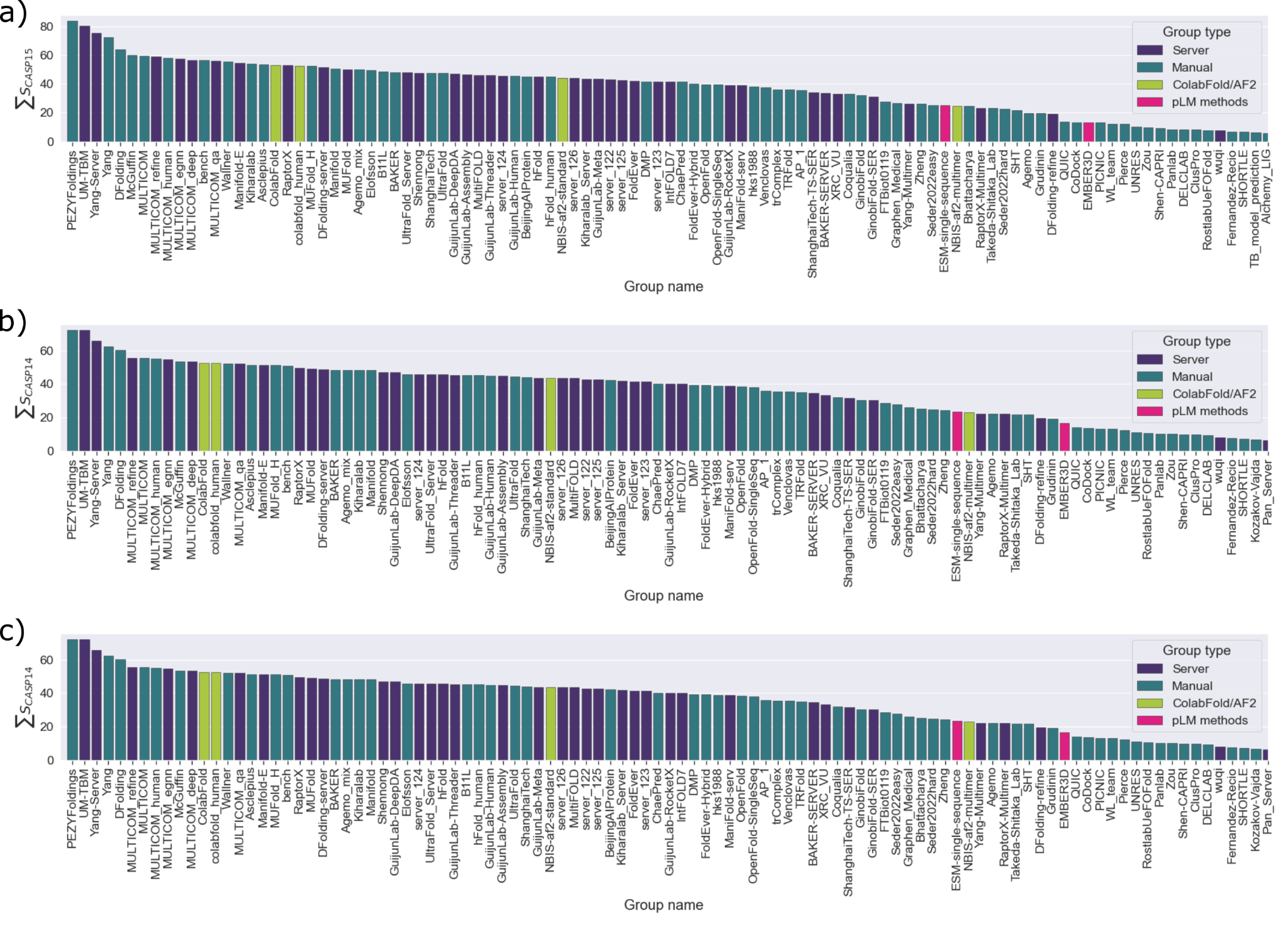
Supp Fig 1

Cumulative group ranking on 109 CASP15 evaluation units. according to the CASP15 score (a) and those of CASPs 14 (b) and 13 (c). Groups are colour-coded indigo for ‘Server’, i.e. a purely automated modelling protocol, and teal for ‘Manual’ where human intervention is allowed. Pure AlphaFold 2 comparison runs based on the original DeepMind protocol or its ColabFold version are shown in green. Pink is used for the three groups employing protein language model methods.

###
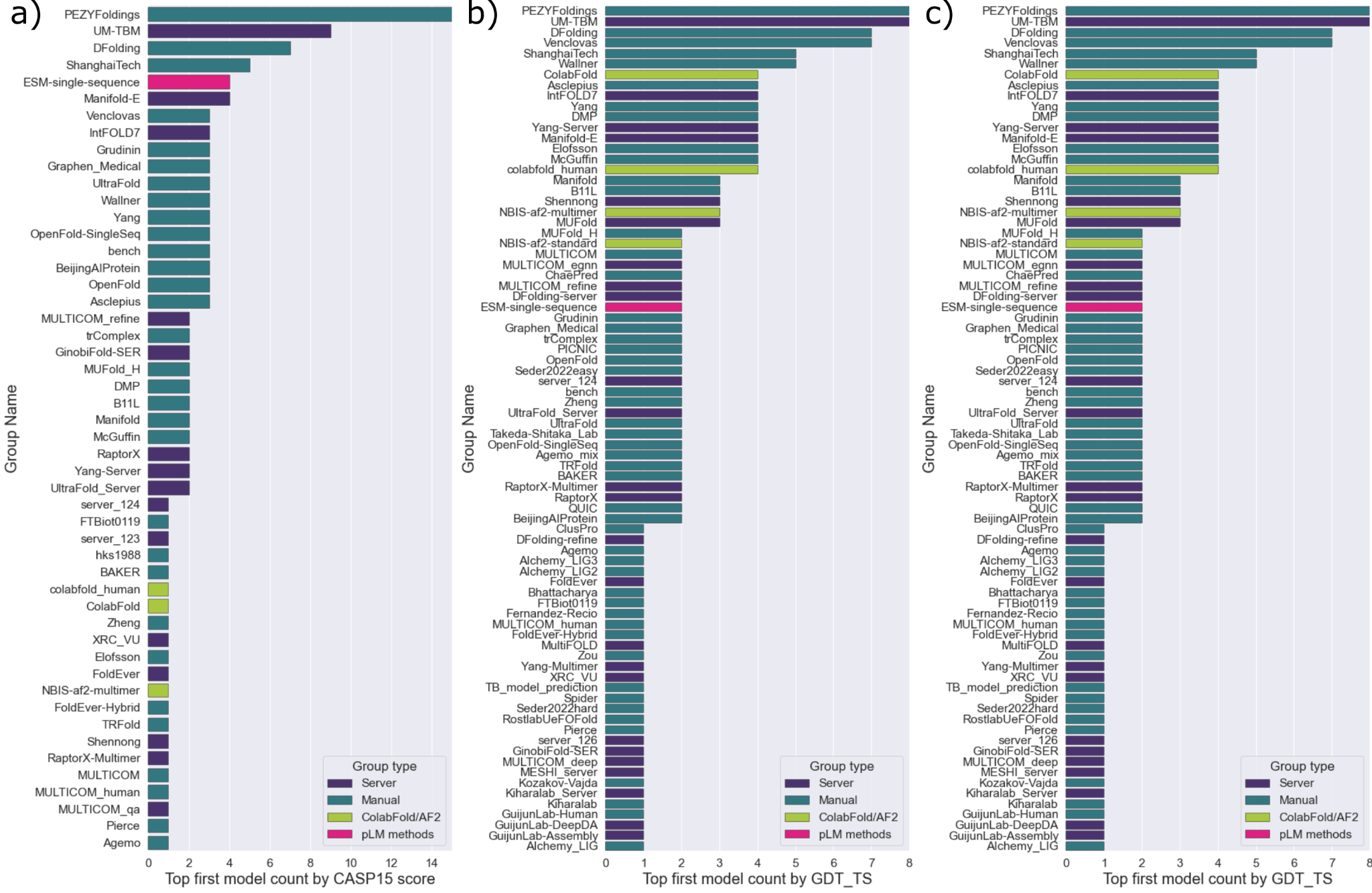
Supp Fig 2

Number of targets for which each group produced the best model by (a) S_CASP15_, (b) GDT_TS and (c) GDT_HA


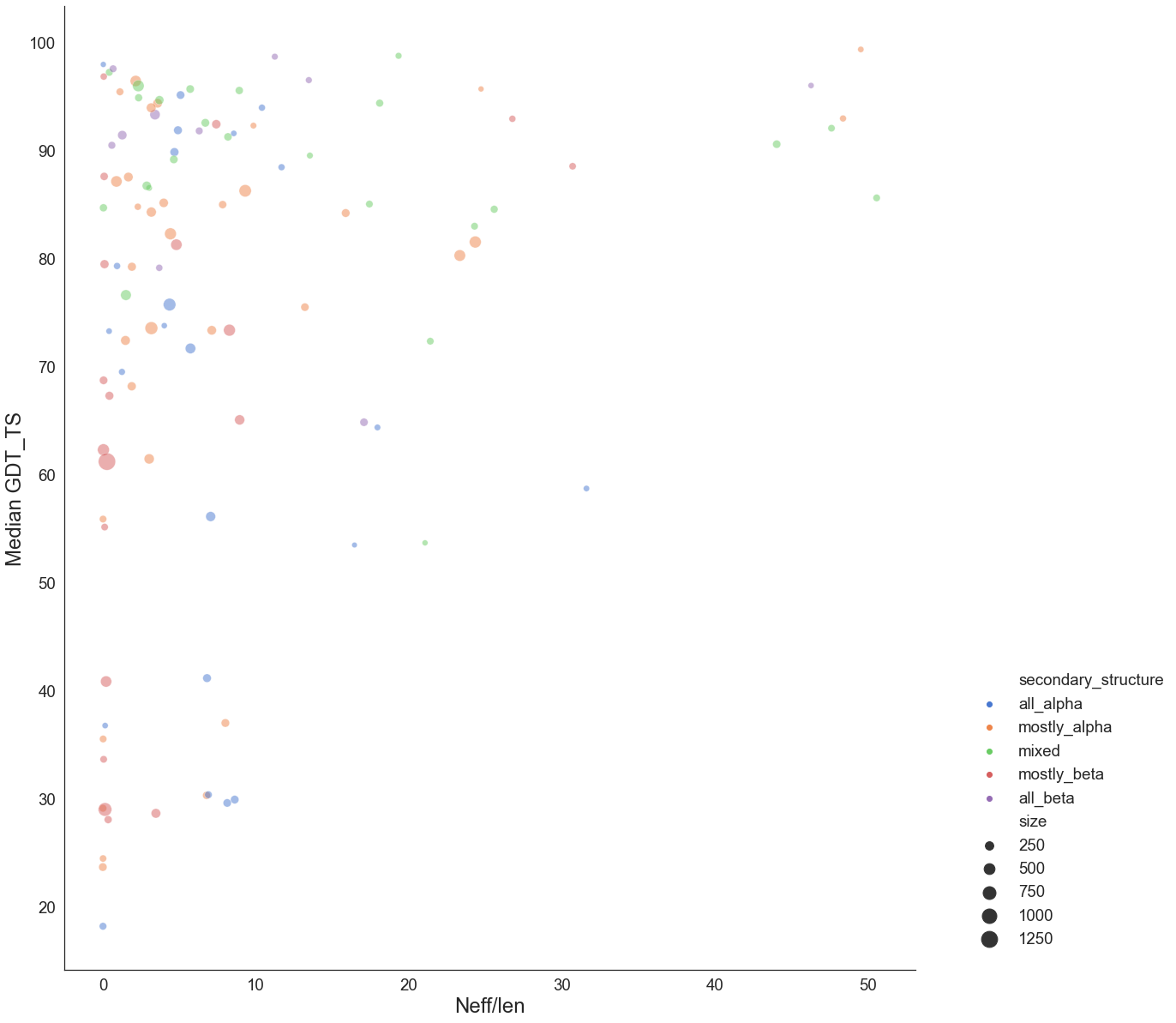


### Supp Fig 3

Scatter plot of GDT_TS versus Target Neff/Target Length for all groups. The scatter points are coloured by secondary structure and the size of the points correspond to the size of the target.


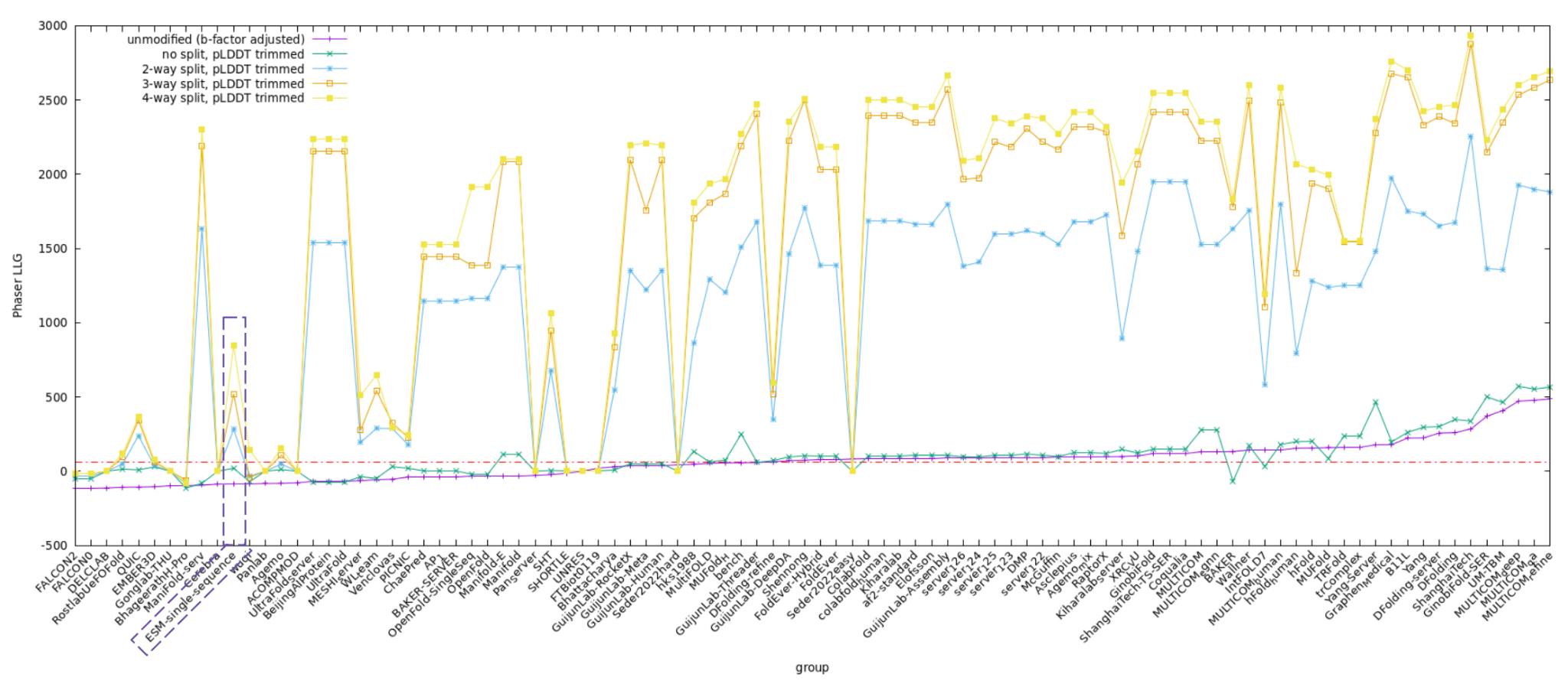


### Supp Fig 4

Phaser LLG scores where model 1 from all of the groups has been aligned to the target T1145. Results are presented for the unsplit model apart from pLDDT-to-B-factor conversion (no removal of residues), the unsplit model with residues scoring pLDDT<70 removed, and for the model split into 2, 3 and 4 parts using the Slice’n’Dice method, also with residues having pLDDT<70 removed. Cases where the LLG fails to improve with splitting are where the group has used something other than the standard pLDDT for residue confidence scoring and the conversion to B-factor and removal of low confidence residues was not performed.


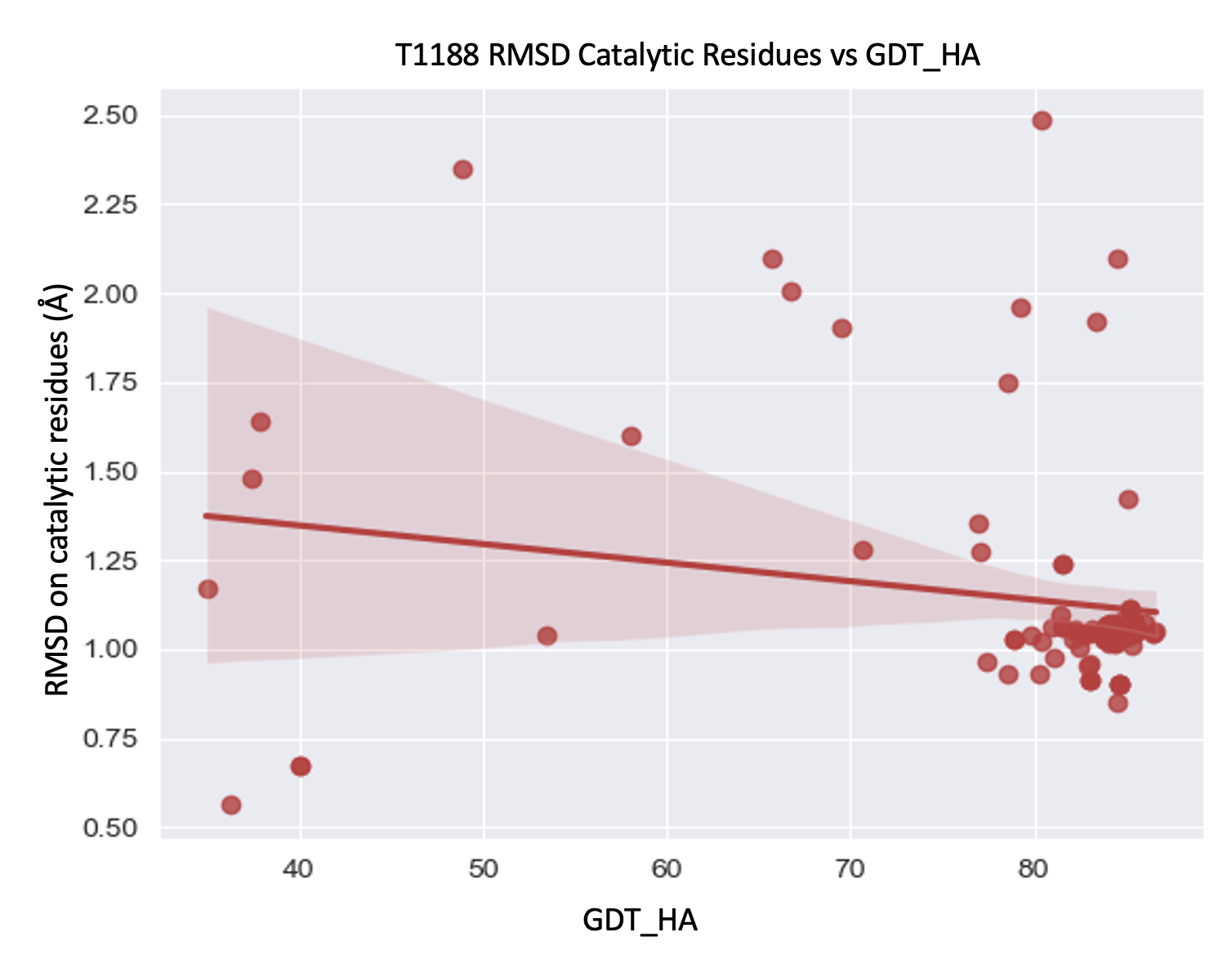

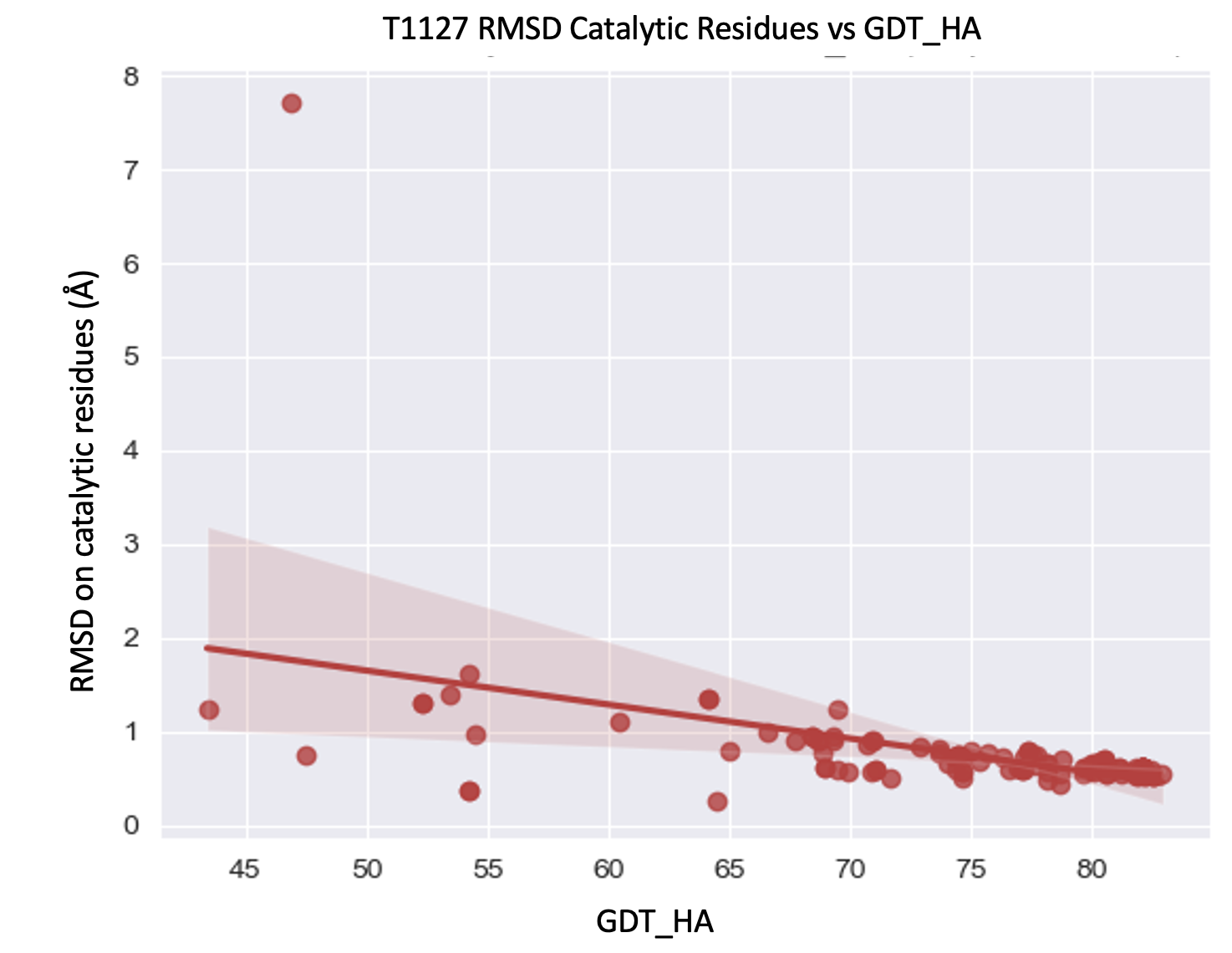

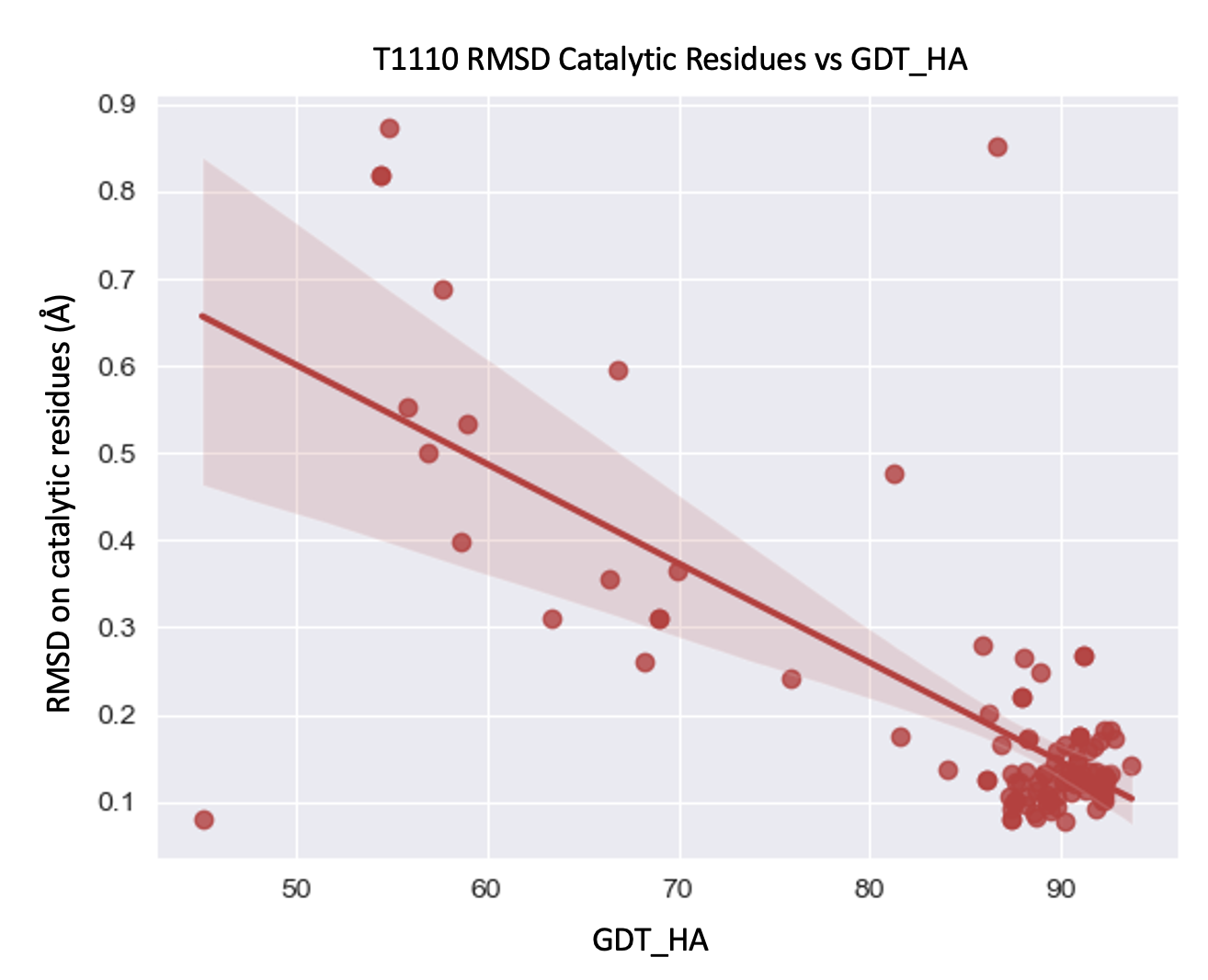


### Supp Fig 5.

RMSD of catalytic residues. Global accuracy and the accuracy of functional features are only weakly correlated, as shown here by the RMSD on catalytic residues vs GDT_HA for T1188, T1127 and T1110.

### Supplementary table 1.

Characteristics of the CASP15 single chain targets with available diffraction data

| Target | No. chains | Resolution (Å) | Space group | No. residues/chain |
| --- | --- | --- | --- | --- |
| T1104 | 1 | 1.8 | C2 | 118 |
| T1113 | 4 | 2.63 | P1211 | 194 |
| T1122 | 1 | 1.7 | P3221 | 242 |
| T1125 | 1 | 3.1 | P4122 | 1201 |
| T1145 | 2 | 2.2 | P212121 | 636 |
| T1150 | 2 | 1.86 | C2221 | 371 |
| T1159 | 2 | 1.84 | P212121 | 161 |
| T1162 | 2 | 1.52 | P21 | 197 |
| T1163 | 1 | 1.4 | P41212 | 192 |
| T1174 | 2 | 2.5 | P63 | 339 |
| T1175 | 2 | 2 | C121 | 313 |
| T1176 | 2 | 2 | C121 | 171 |
| T1177 | 1 | 2 | P212121 | 224 |
| T1181 | 3 | 2.3 | P65 | 689 |
| T1187 | 8 | 2 | P21 | 167 |
| T1188 | 1 | 1.85 | P41212 | 631 |
| T1194 | 2 | 1.93 | P41212 | 169 |
